# Cannabis use disorder contributes to cognitive dysfunction in Veterans with traumatic brain injury

**DOI:** 10.3389/fneur.2024.1261249

**Published:** 2024-01-16

**Authors:** Aryan Esmaeili, Clara Dismuke-Greer, Terri K. Pogoda, Megan E. Amuan, Carla Garcia, Ariana Del Negro, Maddy Myers, Eamonn Kennedy, David Cifu, Mary Jo Pugh

**Affiliations:** ^1^Health Economics Resource Center (HERC), Ci2i, VA Palo Alto Health Care System, Menlo Park, CA, United States; ^2^Center for Healthcare Organization and Implementation Research, VA Boston Healthcare System, Boston, MA, United States; ^3^Boston University School of Public Health, Boston, MA, United States; ^4^Informatics, Decision-Enhancement, and Analytic Sciences Center of Innovation, VA Salt Lake City Health Care System, Salt Lake City, UT, United States; ^5^Division of Epidemiology, Department of Internal Medicine, University of Utah School of Medicine, Salt Lake City, UT, United States; ^6^Department of Physical Medicine and Rehabilitation, School of Medicine, Virginia Commonwealth University, Richmond, VA, United States

**Keywords:** traumatic brain injury, cannabis use disorder, dementia, Veterans, Cox proportional hazards model

## Abstract

**Background:**

While emerging evidence supports a link between traumatic brain injury (TBI) and progressive cognitive dysfunction in Veterans, there is insufficient information on the impact of cannabis use disorder (CUD) on long-term cognitive disorders. This study aimed to examine the incidences of cognitive disorders in Veterans with TBI and CUD and to evaluate their relationship.

**Methods:**

This retrospective cohort study used the US Department of Veterans Affairs and Department of Defense administrative data from the Long-term Impact of Military-Relevant Brain Injury Consortium-Chronic Effects of Neurotrauma Consortium Phenotype study. Diagnoses suggesting cognitive disorders after a TBI index date were identified using inpatient and outpatient data from 2003 to 2022. We compared the differential cognitive disorders incidence in Veterans who had the following: (1) no CUD or TBI (control group), (2) CUD only, (3) TBI only, and (4) comorbid CUD+TBI. Kaplan-Meier analyses were used to estimate the overall cognitive disorders incidence in the above study groups. The crude and adjusted Cox proportional hazards models were used to estimate crude and adjusted hazard ratios (HRs) for cognitive disorders.

**Results:**

A total of 1,560,556 Veterans [82.32% male, median (IQR) age at the time of TBI, 34.51 (11.29) years, and 61.35% white] were evaluated. The cognitive disorder incidence rates were estimated as 0.68 (95% CI, 0.62, 0.75) for CUD only and 1.03 (95% CI, 1.00, 1.06) for TBI only per 10,000 person-months of observations, with the highest estimated cognitive disorder incidence observed in participants with both TBI and CUD [1.83 (95% CI, 1.72, 1.95)]. Relative to the control group, the highest hazard of cognitive disorders was observed in Veterans with CUD+TBI [hazard ratio (HR), 3.26; 95% CI, 2.91, 3.65], followed by those with TBI only (2.32; 95 CI%, 2.13, 2.53) and with CUD (1.79; 95 CI%, 1.60, 2.00). Of note, in the CUD only subgroup, we also observed the highest risk of an early onset cognitive disorder other than Alzheimer's disease and Frontotemporal dementia.

**Discussion:**

The results of this analysis suggest that individuals with comorbid TBI and CUD may be at increased risk for early onset cognitive disorders, including dementia.

## Introduction

Individuals with traumatic brain injury (TBI), independent of severity, are at increased risk for dementia (1) a neurodegenerative disorder that is characterized by a decline in one or more cognitive domains (2) and profoundly affects mortality, quality of life, caregiver stress, and economic burden (3). The risk of dementia is of particular concern for Veterans with TBI since they frequently present with other associated risk factors for dementia, including post-traumatic stress disorder (PTSD), depression, and sleep impairment that may compound risk and accelerate neurodegenerative processes (4, 5). TBI has been a central focus of morbidity in recent war efforts, as nearly 20% of the more than 2.5 million deployed U.S. military Service Members and Veterans (SMVs) since 2003 sustained at least one TBI (6, 7). Importantly, more than 80% of the TBIs are mild in severity (mTBI) and up to 8% of all Veterans who have sustained TBI are expected to have persistent symptoms related to the event more than 6 months post-injury (8, 9). Difficulty with cognitive, affective, somatosensory, and vestibular symptoms are common post-TBI complaints (10, 11). Post-9/11 combat-deployed service members are at risk of single and repetitive blast and non-blast injuries, in particular mild TBIs (12). Although yet to be fully defined, the mechanisms by which TBI promotes neurodegeneration may be modulated by an array of processes manifesting from insult related neuropathological changes that may be further exacerbated by repetitive injury (13, 14). A history of TBI exposure may also accelerate the time to dementia diagnosis (15), evidenced by a recent study showing an increased risk for early-onset dementia in young post-9/11 Veterans with prior TBI (16).

No study has demonstrated the beneficial effects of smoking marijuana (17). To date, the United States Food and Drug Administration (FDA) has not recommended cannabis for the treatment of any disease or condition (18). Cannabis use disorder (CUD) is defined as problematic marijuana use that causes impairment or distress, without necessarily leading to addiction (19, 20). Zehra et al. (21) suggested that CUD possesses addictive properties akin to other drugs of abuse. Despite the lack of efficacy, cannabis is frequently used to self-treat a wide array of symptoms and conditions, including those associated with persistent post-concussion symptoms (e.g., chronic pain, headache, insomnia, anxiety, irritability, etc.) (22–25). Owing to expanded legalization, lower perceptions of risk, and the absence of established medication regimens (26), the use of cannabis for symptom management following TBI has likely increased (25, 27) in parallel with growing trends in overall use in both the general U.S. population (28) and in Veterans (29–32). Cannabinoids may regulate some of the processes that lead to neurodegeneration (33), and therefore may be useful in the treatment of neurodegenerative dementias such as Alzheimer's disease (AD), in particular for symptoms of agitation (34, 35). However, to date, systematic reviews have noted that available data evaluating cannabinoids for the treatment of dementia progression are insufficient to draw clear conclusions (36, 37). Additionally, studies have shown that cannabis use acutely impairs cognitive functions including attention, concentration, episodic memory, and associative learning in a dose-dependent fashion (38, 39). As observed in some types of dementia, structural changes, such as decreases in regional brain volume in the hippocampus, amygdala, and striatum have also been linked to heavy, chronic cannabis use (40–45). The existence of these non-conclusive and contradictory studies on effectiveness of cannabis on dementia treatment warrant further study. The objective of this study was to examine the association of CUD in the emergence of cognitive disorders in Post-9/11 Veterans diagnosed with TBI.

## Methods

### Participants and data source

The cohort for this retrospective analysis included participants from the Long-term Impact of Military-Relevant Brain Injury Consortium–Chronic Effects of Neurotrauma Consortium (LIMBIC-CENC) Phenotype study. As described in detail previously (46), this is a large cohort of Post-9/11 active duty and veteran U.S. military persons who received care in the Department of Defense (DoD) for at least 3 years, including those exposed and unexposed to TBI(s). Data for this study included healthcare data during deployment [e.g., DoD Trauma Registry (DoDTR) and Theater Management Data Store (TMDS)], DoD, VA, and Non-VA community inpatient and outpatient data. To ensure accurate TBI status and sufficient data to identify cognitive disorder, we included only those participants who also had 2 years of care in the Veterans Health Administration (VHA) during the study period. The research protocol was reviewed and approved by the University of Utah and Stanford institutional review board (IRB) and was conducted in accordance with all applicable federal regulations.

### Measures and outcomes

#### Development of study groups

**Figure 1:**
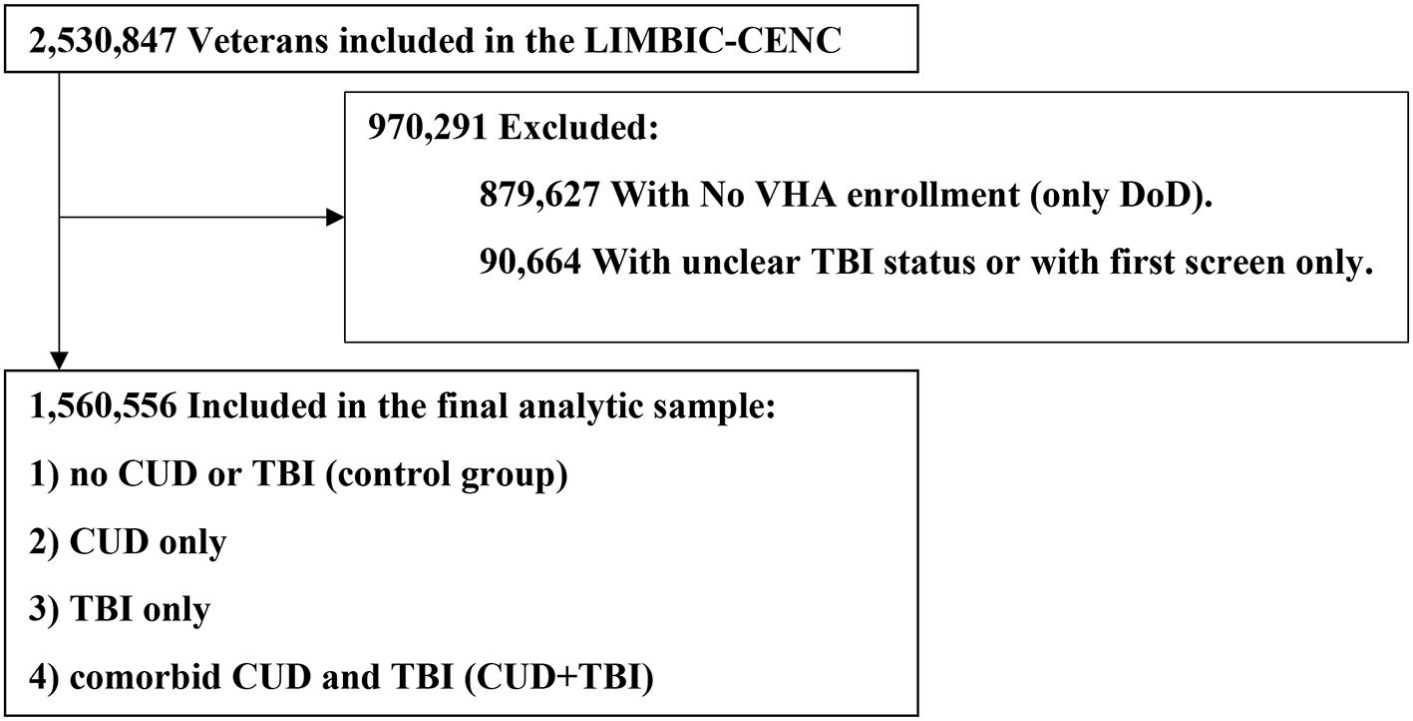
Study flow chart for impact of cannabis on cognitive disorder in Veteran patients with TBI. CUD, Cannabis Use Disorder; DoD, Department of Defense; LIMBIC-CENC, Long-term Impact of Military-Relevant Brain Injury Consortium-Chronic Effects of Neurotrauma Consortium; TBI, Traumatic Brain Injury; VHA, Veterans Health Administration.

We used a hierarchical approach to identify TBI by prioritizing data from DoDTR and TMDS (Glasgow Coma Scale score, Abbreviated Injury Severity Score, and ICD-9-CM and ICD-10-CM codes), followed by self-reported data from the comprehensive TBI evaluation (CTBIE) data collected in the process of clinical care loss of consciousness (mild, ≤ 30 min; moderate to severe, >30 min), alteration of consciousness or posttraumatic amnesia (mild <24 h; moderate to severe, ≥24 h 16), and ICD-9/10-CM diagnosis codes from the Armed Forces Health Surveillance Division algorithm (47). The *index date* for TBI was the first date of diagnosis or the date of the CTBIE assessment; for those with more than one TBI documented we used the date of the most severe TBI. Veterans who did not enroll in VHA and did not complete the initial VA screening for TBI were excluded from the study (Figure 1). For those without TBI we calculated simulated TBI index dates using a Monte Carlo simulation to generate age-correlated index dates (16). To establish comparable analysis time windows across groups, it was necessary to assign a simulated index date to each individual in the non-TBI group. The simulated index dates were drawn at random from the real distribution of injury dates. To further refine this approach, the simulated index dates were only sampled from a subset of those in the TBI group who were of a similar age as the TBI negative individual (within 5 years) (16).

Cognitive disorder was indicated by ICD-9/10 diagnosis codes used to identify dementia by VHA Geriatrics and Extended Care (Supplementary Table 3); ascertainment required a single ICD-9/10 dementia diagnosis code after TBI index date through September 11, 2022. Since prior research indicates that these diagnoses are not accurate for individuals under age 65 (48, 49), we classified these diagnoses simply as a “cognitive disorder.” To increase confidence in confirmed dementia cases, we performed a sensitivity analysis on the cohort with at least two ICD-9/10 codes for dementia diagnosis and in the subgroup of 1) early onset of dementia (EOD) (16), consisting of AD (ICD10 = G30.0), and Frontotemporal dementia (FTD) and 2) non-EOD subgroups consisting of early onset cognitive disorder other than AD and FTD.

#### Primary outcome

The primary outcome, time from TBI index date to cognitive disorder diagnosis, was calculated using the TBI/simulated index date and the first documented diagnosis indicating cognitive disorder. To evaluate the associations among CUD and cognitive disorder emergence following TBI, we categorized the cohort into four groups: (1) Neither CUD nor TBI (control group), (2) TBI only, (3) CUD only, and (4) comorbid CUD and TBI (CUD+TBI). Cannabis CUD was identified using ICD-9 (304.3: Cannabis dependence, and 305.2: Non-dependent cannabis abuse), and ICD-10 codes (F12: Cannabis-related disorders including F12.1 Cannabis abuse, F12.2 Cannabis dependence, and F12.9 Cannabis use, unspecified) for any ICD-9/10 diagnoses after TBI index date.

#### Covariates

Sociodemographic and military characteristics, including biological sex, age at index date, race/ethnicity, education, marital status, branch, rank, rural/urban location of Veteran residence, and VA service-connected disability group were obtained from the VA DoD Identity Repository (VADIR; FY00-FY19) at the time of military discharge.

#### Clinical characteristics

We identified comorbid conditions using ICD-9 and 10 codes obtained from the VA Corporate Data Warehouse (CDW) and the DoD and VA Infrastructure for Clinical Intelligence (DaVINCI). Conditions were identified using algorithms provided in Supplementary Table 3 when individuals had one or more ICD-9 and 10 diagnoses between TBI index date to September 11, 2022. We identified the Medication-assisted treatment (MAT) after the TBI index date using the algorithm provided by VA Pharmacy Benefits Management Services (Supplementary Table 3). The U.S. district/region is identified using the VA medical center (stations) that was assigned based on the Veteran home address (Veteran residence).

#### Statistical analysis

Descriptive analyses of demographic characteristics and risk behaviors from baseline data by CUD and TBI status were conducted. We estimated the cognitive disorder incidence rates (IRs) using Kaplan-Meier methods for the overall cohort and for each of the four groups. Participants were censored at the date of their last health care system encounter or September 11, 2022 (whichever came first). We used Cox proportional hazards models to calculate the crude and adjusted CUD and TBI-specific hazard ratio (HR) for cognitive disorder incidence using CUD+TBI as the main exposure, controlling for sociodemographic and clinical characteristics. To increase confidence in confirmed dementia cases, we performed a sensitivity analysis by re-running the Cox proportional hazards models in the EOD and Non-EOD cohort (**Table 2**). All analyses were conducted using Stata version 17 (StataCorp LP, College Station, TX).

## Results

### Sociodemographic and clinical characteristics

A total of 1,560,556 Veterans were included in the analysis and stratified by TBI and CUD status. Table 1 presents some of the key demographic and health characteristics of each of the four groups. A fuller range of these variables may be seen in Supplementary Table 1, which also includes standardized mean differences between the clinical and population differences by TBI and CUD status. The median (IQR) age at the time of TBI was 34.51 (11.29) years. Veterans with CUD+TBI tended to be male, with a high school education or less, were enlisted in the Army, and had higher service-connected disability percentages compared with the other 3 groups (Table 1). The TBI-CUD group also had higher rates of diagnoses for headache, other chronic pain, participation in MAT programs, mental health conditions [i.e., severe mental illness (such as schizophrenia, Bipolar II disorder), depression, PTSD, personality disorder, anxiety, insomnia], and alcohol, opioid, and other substance use disorders compared with the other groups.

**Table 1:** Demographic and clinical characteristics of veterans by CUD and TBI status (*N* = 1,560,556).

	**Control *N* (%)**	**TBI only *N* (%)**	**CUD only *N* (%)**	**CUD + TBI *N* (%)**	**Total *N* (%)**
Overall	1,12,4686 (72.07)	3,45,896 (22.16)	48,100 (3.08)	41,874 (2.68)	1,560,556 (100)
Male	9,02,228 (80.22)	3,04,369 (87.99)	39,783 (82.71)	38,243 (91.33)	1,284,623 (82.32)
Age at TBI (Mean ± SD)	35.36 (11.60)	33.30 (10.42)	28.43 (8.07)	28.58 (7.29)	34.51 (11.29)
Race/ethnicity (White)	6,84,525 (60.86)	2,20,561 (63.77)	26,501 (55.1)	25,796 (61.6)	9,57,383 (61.35)
Black or African American	2,32,517 (20.67)	54,881 (15.87)	13,172 (27.38)	7,211 (17.22)	3,07,781 (19.72)
Hispanic or Latino	1,15,010 (10.23)	35,771 (10.34)	4,353 (9.05)	3,629 (8.67)	1,58,763 (10.17)
Other	86,198 (7.66)	33,403 (9.66)	3,901 (8.11)	5,150 (12.3)	1,28,652 (8.24)
Unknown	6,436 (0.57)	1,280 (0.37)	173 (0.36)	88 (0.21)	7,977 (0.51)
Education (college and above)	3,46,404 (30.8)	83,513 (24.14)	5,730 (11.91)	4,491 (10.73)	4,40,138 (28.2)
High school and less	7,74,755 (68.89)	2,61,783 (75.68)	42,282 (87.9)	37,330 (89.15)	1,116,150 (71.52)
Unknown	3,527 (0.31)	600 (0.17)	88 (0.18)	53 (0.13)	4,268 (0.27)
Marital status (not married)	5,44,334 (48.4)	1,62,840 (47.08)	33,069 (68.75)	27,000 (64.48)	7,67,243 (49.16)
Married	5,79,138 (51.49)	1,82,875 (52.87)	15,001 (31.19)	14,857 (35.48)	7,91,871 (50.74)
Unknown	1,214 (0.11)	181 (0.05)	30 (0.06)	17 (0.04)	1,442 (0.09)
Branch (air force)	2,08,515 (18.54)	34,084 (9.85)	5,661 (11.77)	2,487 (5.94)	2,50,747 (16.07)
Army	5,10,432 (45.38)	2,06,662 (59.75)	26,205 (54.48)	28,100 (67.11)	7,71,399 (49.43)
Marines	1,61,454 (14.36)	61,554 (17.8)	6,953 (14.46)	6,957 (16.61)	2,36,918 (15.18)
Navy/coast guard	2,43,312 (21.63)	43,433 (12.56)	9,275 (19.28)	4,328 (10.34)	30,0348 (19.25)
Other	973 (0.09)	163 (0.05)	6 (0.01)	2 (0)	1,144 (0.07)
Rank (enlisted)	1,004,715 (89.34)	3,21,134 (92.85)	47,300 (98.34)	41,262 (98.54)	1,414,411 (90.64)
Officer	1,05,095 (9.34)	20,969 (6.06)	672 (1.4)	508 (1.21)	1,27,244 (8.15)
Warrant	14,812 (1.32)	3,764 (1.09)	124 (0.26)	102 (0.24)	18,802 (1.2)
Rurality (Rural)	3,13,229 (27.85)	1,09,710 (31.72)	12,344 (25.66)	12,941 (30.9)	4,48,224 (28.72)
Urban	8,06,537 (71.71)	2,35,011 (67.94)	35,677 (74.17)	28,840 (68.87)	1,106,065 (70.88)
Unknown	4,920 (0.44)	1,175 (0.34)	79 (0.16)	93 (0.22)	6,267 (0.4)
VA SCD None/0%	2,54,515 (22.63)	39,619 (11.45)	10,384 (21.59)	5,060 (12.08)	30,9578 (19.84)
10-40 percent	1,85,725 (16.51)	24,283 (7.02)	4,943 (10.28)	1,831 (4.37)	2,16,782 (13.89)
≥50 percent	6,84,446 (60.86)	2,81,994 (81.53)	32,773 (68.14)	34,983 (83.54)	1,034,196 (66.27)
District (North Atlantic)	2,46,134 (21.89)	71,638 (20.71)	9,963 (20.71)	8,365 (19.98)	3,36,100 (21.54)
Southeast	2,29,057 (20.37)	67,119 (19.4)	10,061 (20.92)	7,832 (18.7)	3,14,069 (20.13)
Midwest	2,17,771 (19.36)	70,310 (20.33)	9,437 (19.62)	8,701 (20.78)	3,06,219 (19.62)
Continental	2,26,839 (20.17)	73,873 (21.36)	9,917 (20.62)	8,982 (21.45)	3,19,611 (20.48)
Pacific	2,04,855 (18.21)	62,948 (18.2)	8,720 (18.13)	7,994 (19.09)	2,84,517 (18.23)
Headache	2,82,990 (25.16)	1,97,752 (57.17)	14,821 (30.81)	25,226 (60.24)	5,20,789 (33.37)
Other chronic pain	8,84,584 (78.65)	3,14,294 (90.86)	41,360 (85.99)	39,197 (93.61)	1,279,435 (81.99)
EOD (AD and FTD disease)	845 (0.08)	527 (0.15)	38 (0.08)	59 (0.14)	1,469 (0.09)
MAT (recent)	29,563 (2.63)	19,292 (5.58)	10,485 (21.8)	11,650 (27.82)	70,990 (4.55)
Severe mental illness	1,28,519 (11.43)	82,841 (23.95)	23,283 (48.41)	25,302 (60.42)	2,59,945 (16.66)
Depression	3,89,215 (34.61)	1,90,748 (55.15)	36,809 (76.53)	35,197 (84.05)	6,51,969 (41.78)
PTSD	2,92,631 (26.02)	2,25,294 (65.13)	28,998 (60.29)	36,407 (86.94)	5,83,330 (37.38)
Personality disorder	22,613 (2.01)	15,817 (4.57)	8,488 (17.65)	10,123 (24.17)	57,041 (3.66)
Alcohol use disorder	2,09,531 (18.63)	1,21,974 (35.26)	34,111 (70.92)	34,672 (82.8)	4,00,288 (25.65)
Opioid use disorder	24,459 (2.17)	20,824 (6.02)	14,697 (30.56)	18,720 (44.71)	78,700 (5.04)
Other drug use disorder	28,869 (2.57)	24,150 (6.98)	28,052 (58.32)	27,556 (65.81)	1,08,627 (6.96)
Nicotine use disorder	1,78,754 (15.89)	93,409 (27)	20,290 (42.18)	23,682 (56.56)	3,16,135 (20.26)
Anxiety	3,63,430 (32.31)	1,74,680 (50.5)	32,448 (67.46)	31,843 (76.04)	6,02,401 (38.6)
Insomnia	2,07,220 (18.42)	1,17,128 (33.86)	10,998 (22.86)	16,704 (39.89)	3,52,050 (22.56)
Memory loss	14,928 (1.33)	56,855 (16.44)	1,072 (2.23)	8,023 (19.16)	80,878 (5.18)
CHF	20,709 (1.84)	7,384 (2.13)	801 (1.67)	864 (2.06)	29,758 (1.91)
Cardiac disease	1,12,527 (10.01)	50,198 (14.51)	5,921 (12.31)	8,178 (19.53)	1,76,824 (11.33)
Stroke	15,412 (1.37)	13,779 (3.98)	588 (1.22)	1,725 (4.12)	31,504 (2.02)
Convulsions disorders	91,835 (8.17)	87,675 (25.35)	11,772 (24.47)	20,070 (47.93)	2,11,352 (13.54)
CKD	19,286 (1.71)	6,535 (1.89)	860 (1.79)	844 (2.02)	27,525 (1.76)

SCD, Service Connected Disability; TBI, Traumatic Brain Injury; CUD, Cannabis Use Disorder; MAT, Medication-Assisted Treatment; CHF, Congestive Heart Failure; CKD, Chronic Kidney Disease; PTSD, Post-Traumatic Stress Disorder; EOD, Early Onset Dementia; AD, Alzheimer's disease; FTD, Frontotemporal dementia. The statistical difference is significant for all variables (p <0.005).

### Cognitive disorder IR and hazard ratio by TBI and CUD status

**Figure 2:**
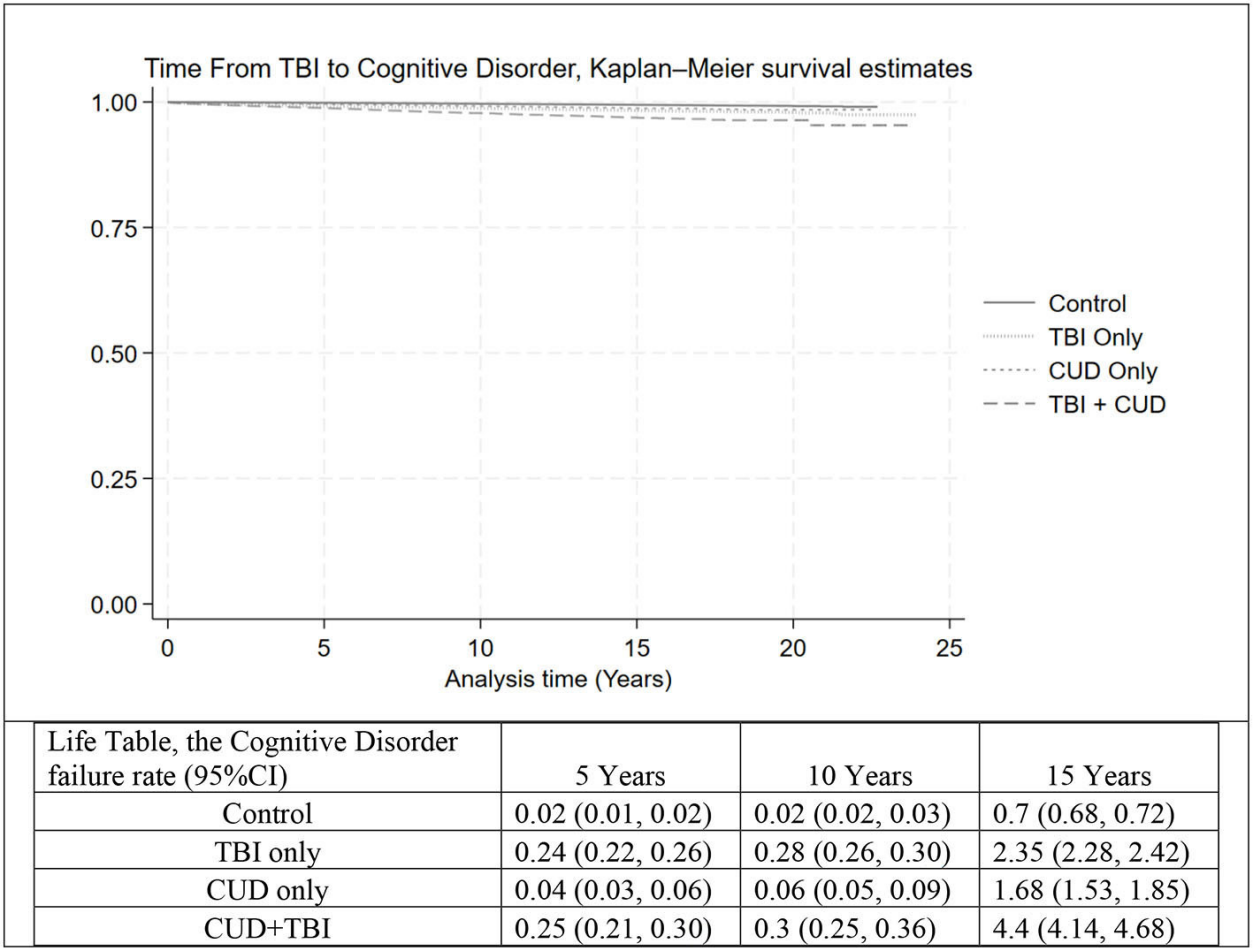
The time from TBI (or TBI index date in the control group) to cognitive disorder, Kaplan–Meier survival estimate and life table, in four groups: Control, TBI only, CUD only, and CUD+TBI. TBI, Traumatic Brain Injury; CUD, Cannabis Use Disorder.

The cognitive disorder IR and corresponding HR by TBI and CUD status are shown in Table 2. Overall, we identified 9,844 Veterans with a history of any type of cognitive disorder. The overall cognitive disorder IR was estimated as 0.52 (95% CI: 0.51, 0.53) per 10,000 person months of observations (PMO). After controlling for all demographic and risk factors, the hazard of cognitive disorder was 2.32 (95% CI: 2.13, 2.53), 1.79 (95% CI: 1.60, 2.00), and 3.26 (95% CI: 2.91, 3.65) for Veterans with TBI only, CUD only, and CUD+TBI, respectively, compared to the control group. Figure 2 shows the time from TBI to cognitive disorder by subgroup. Despite a very low incidence of cognitive disorder in our cohort, the risk of cognitive disorder was significantly higher in Veterans with CUD+TBI. The cognitive disorder rate was 0.25%, 0.30%, and 4.4% at 5, 10, and 15 years after TBI, respectively, in Veterans with CUD + TBI. After controlling for all demographic and risk factors, the modifying effect (interaction term) between CUD and TBI on the progression of dementia was <22% the expected rate for the combined risks of TBI and CUD [Supplementary Table 2, HR = 0.78 (95% CI: 0.69, 0.89)] in Veterans diagnosed with TBI.

**Table 2:** Cognitive disorder incidence rate (overall and by TBI and CUD status), and hazard ratio of dementia by CUD and TBI status.

	**Person-time**	**Failures (Documented cognitive disorder)**	**IR (95% CI) per 10000 PMO**	**Crude**	Adjusted model^*^
				**HR (95% CI)**	**HR (95% CI)**
**All types of cognitive disorder**					
Overall	190,800,000	9,844	0.52 (0.51, 0.53)		
Control	136,600,000	4,053	0.30 (0.29, 0.31)	Ref	Ref
TBI only	4,255,8896	4,381	1.03 (1.00, 1.06)	3.47 (3.33, 3.62)	2.32 (2.13, 2.53)
CUD only	6213722.8	423	0.68 (0.62, 0.75)	2.31 (2.09, 2.55)	1.79 (1.60, 2.00)
CUD+TBI	5386415.6	987	1.83 (1.72, 1.95)	6.21 (5.79, 6.65)	3.26 (2.91, 3.65)
**EOD (AD and FTD disease)**					
Overall	191,300,000	1,053	0.06 (0.05, 0.06)		
Control	136,800,000	646	0.05 (0.04, 0.05)	Ref	Ref
TBI only	42,866,265	354	0.08 (0.07, 0.09)	1.75 (1.54, 1.99)	1.75 (1.3, 2.35)
CUD only	6237732.1	20	0.03 (0.02, 0.05)	0.68 (0.44, 1.07)	1.49 (0.93, 2.39)
CUD+TBI	5458252.4	33	0.06 (0.04, 0.09)	1.29 (0.91, 1.83)	2.81 (1.74, 4.53)
**All other early onset cognitive disorder (Non-EOD)**					
Overall	191,100,000	4,307	0.23 (0.22, 0.23)		
Control	136,700,000	1,529	0.11 (0.11, 0.12)	Ref	Ref
TBI only	42,696,148	2,195	0.51 (0.49, 0.54)	4.61 (4.32, 4.92)	3.04 (2.68, 3.44)
CUD only	6228235.2	157	0.25 (0.22, 0.29)	2.27 (1.93, 2.68)	1.84 (1.54, 2.20)
CUD+TBI	5426337.5	426	0.79 (0.71, 0.86)	7.08 (6.36, 7.88)	3.95 (3.33, 4.67)

HR, Hazard Ratio; IR, Incidence Rate; CI, Confidence Interval; EOD, Early Onset Dementia; TBI, Traumatic Brain Injury; CUD, Cannabis Use Disorder; PMO, Person Months of Observations; Ref, Reference. The covariates included in the adjusted model: CUD, TBI, sex, age at the time of TBI, TBI severity, race, education, marital status, branch, rank, rurality, service-connected disability groups, District, Headache, Chronic Pain, MAT (recent), Oncology, Severe Mental Illness, Depression, PTSD, Personality Disorder, Alcohol Use Disorder, Opioid Use Disorder, Other SUD, Nicotine Use disorder, anxiety, insomnia, CHF, Perivascular disease, Cardiac disease, Stroke, Diabetes Mellitus (DM), DM with complications, convulsions disorders, Neurologic disorder (No Convulsions disorders), Liver Disease, CKD, and death. ^*^The covariates included in the adjusted model.

### EOD vs. non-EOD

Among the 9,844 Veterans with an assigned diagnosis of cognitive disorder, 5,360 were identified as early onset cognitive disorder with at least 2 documented dementia diagnoses (1,053 and 4,307 Veterans with EOD and non-EOD, respectively, Table 2). The crude and adjusted HRs of the CUD+TBI, CUD only, and TBI only groups, relative to the control groups, were lower in the EOD subgroup relative to the Non-EOD group. We did not observe any significant differences in the hazard of EOD between the CUD only and control groups.

### EOD and non-EOD among Veterans with TBI

We examined factors related to EOD and Non-EOD development among Veterans with TBI, after adjusting for select variables: TBI severity, sex, race/ethnicity, and education, as shown in Table 3. More severe categories of TBI were associated with higher risk of dementia development, as observed with penetrating and moderate/severe TBI; and conversely, less severe TBI, specifically mild TBI and post concussive syndrome (which is indicative of mild TBI), were associated with lower risk of EOD and non-EOD development relative to no TBI. Other factors related to increased risk of EOD were being male, older age at time of TBI, and Hispanic or Latino ethnicity (relative to White). Other factors related to Non-EOD development were being male, older age at time of TBI, Black or African American (relative to White), and having up to a high school education (relative to completing college or higher).

**Table 3:** The impact of selected demographic and clinical characteristics on cognitive disorder (adjusted cox proportional hazards model) among veterans with TBI (*N* = 1,554,319).

	**All**	**EOD**	**Non-EOD**
**TBI severity (No TBI)**	**Ref**	**Ref**	**Ref**
Mild	0.81 (0.75, 0.89)	0.76 (0.56, 1.04)	0.76 (0.67, 0.86)
Moderate/Severe	1.31 (1.19, 1.44)	1.00 (0.70, 1.45)	1.30 (1.13, 1.49)
Penetrating	2.88 (2.6, 3.2)	1.61 (1.09, 2.38)	3.33 (2.89, 3.85)
Post concussive syndrome	0.40 (0.28, 0.57)	0.45 (0.14, 1.46)	0.42 (0.25, 0.69)
**Male**	1.31 (1.23, 1.4)	1.22 (1.01, 1.48)	1.52 (1.38, 1.68)
**Age at the time of TBI**	1.08 (1.08, 1.08)	1.17 (1.16, 1.18)	1.08 (1.07, 1.08)
**Race (White)**	Ref	Ref	Ref
Black or African American	1.04 (0.99, 1.10)	0.99 (0.83, 1.18)	1.11 (1.02, 1.20)
Hispanic or Latino	1.07 (0.99, 1.14)	1.33 (1.08, 1.64)	1.02 (0.91, 1.13)
Other	0.99 (0.92, 1.07)	0.89 (0.69, 1.14)	1.04 (0.93, 1.16)
Unknown	0.93 (0.64, 1.37)	0.40 (0.06, 2.85)	0.89 (0.48, 1.65)
**Education (college or higher)**	Ref	Ref	Ref
High School and Less	1.14 (1.09, 1.20)	1.04 (0.90, 1.20)	1.19 (1.11, 1.29)
Unknown	1.1 (0.81, 1.50)	0.87 (0.43, 1.77)	1.01 (0.62, 1.64)
Alcohol use disorder	1.08 (1.02, 1.14)	0.94 (0.78, 1.15)	1.08 (0.99, 1.18)
Opioid use disorder	0.82 (0.76, 0.88)	0.71 (0.53, 0.94)	0.78 (0.7, 0.87)
Other drug use disorder	1.47 (1.37, 1.58)	1.26 (0.95, 1.67)	1.43 (1.28, 1.59)
Nicotine use disorder	0.91 (0.86, 0.96)	0.82 (0.68, 1.00)	0.90 (0.83, 0.98)

TBI, Traumatic Brain Injury; CUD, Cannabis Use Disorder; EOD, Early Onset Dementia; Ref, Reference.

## Discussion

Among a large cohort of Post-9/11 Veterans, incidence rates of cognitive disorder were highest among those with a history of TBI and concomitant CUD followed by those with TBI only, CUD only, and those without a history of TBI or CUD. Veterans with CUD + TBI had a 3.26 times higher hazard for cognitive disorder compared with those in the control group. Prior studies have established the association between TBI and dementia (1, 50) and potential mechanisms linking the two conditions (14, 51, 52). As expected, Veterans with TBI only had a 2.32 times higher hazard for cognitive disorder compared with those in the control group. While we are not able to assess a dose-response association between CUD and cognitive disorder, we found a higher hazard of cognitive disorder in those with CUD only and CUD+TBI, compared with the control group. Depending on the type and severity, TBI may be exhibited by focal brain damage causing 'shearing and stretching' injuries in cerebral brain tissues (53, 54) or diffuse axonal injury that may involve subcortical and deeper white matter tissues such as the brainstem and corpus callosum (55). Conversely, the distribution of cannabinoids in the brain, regardless of the intake route, occurs after modifying the deleterious effects on the blood–brain barrier (56). Although the brain's blood supply originates in the base of the skull (the brainstem, amygdala, and hypothalamus) and terminates in the cortical area, a previous study demonstrated that cannabis users exhibited significantly increased blood volumes in the frontal, temporal, and cerebellar areas (57).

While our finding is consistent with a previous study indicating higher risk of EOD in Veterans with TBI (16), our data also suggests that CUD is an independent risk factor for cognitive disorder only in the non-EOD group. Compared with the control group, the CUD-only group exhibited a 79% higher hazard for cognitive disorders, primarily driven by the non-EOD subgroup (excluding AD and FTD). A previous systematic review of brain imaging studies among adolescent cannabis users revealed functional and structural evidence of lesions in the frontoparietal, frontolimbic, frontostriatal, and cerebellum regions (58). The results are also consistent with previous studies demonstrating that cannabis use is associated with cognitive functional disorder and bilateral hippocampal and amygdala volume reduction in midlife patients with heavy, chronic cannabis use (40, 45). Our findings indicate AD and FTD are less likely to be observed in the CUD patients, which may be explained by intact cortical areas in cannabis users but need further investigation. Since AD is characteristically a disease of older age (59) and those who were older tended to have lower cannabis quantity use and fewer consequences associated with cannabis use (60, 61), other considerations include possible age-related behavioral changes among those with CUD. Additional factors not measured in these analyses, including social, structural, and biological characteristics, may contribute to dementia susceptibility in Veterans with non-EOD and co-occurring CUD.

The hazard ratio for dementia diagnosis across all categories was more pronounced in individuals with non-EOD, compared with EOD, which could be explained by genetic risk factors and the physiopathology of TBI and CUD in the progression of dementia. The genetic risk factors may pave the way to approach the hypothesis behind the dissimilarity in our EOD vs. non-EOD results. Previous studies addressed the potential roles of several missense mutations and known variant genes in the pathogenesis of early-onset AD (62) and decreased levels of dopaminergic neurotransmitters in patients with AD (63). Conversely, distinct genetic factors for CUD might concurrently initiate underlying pathways related to AD (64). Cannabinoids have been shown to increase mesolimbic dopamine transmission in the short term (65). The risk factors underlying CUD development likely involve multiple genes that interact with each other and the environment, ultimately leading to cognitive disorders. TBI is defined as an impact, penetration, or rapid movement of the brain within the skull and the event can be classified as either impact (direct contact of the head with an object) or non-impact (encountering non-impact forces like blast waves or rapid acceleration and deceleration) (66).

### Limitations

This study has several limitations. The results were restricted to Veterans and based on characteristics and conditions measured and stored in electronic health records (EHRs). Therefore, they may not represent other patient populations. We attempted to account for the difficulties associated with obtaining chronicity and severity of cannabis use by examining both DoD and VA records and limiting cannabis exposures to ICD codes related to CUD. The EHR system in VHA allowed us to identify a CUD diagnosis after the TBI index date, further strengthening the methodology. However, we note that we were not able to quantify the route of cannabis intake (i.e., inhalation vs. ingestion), which is an area for further exploration. While our approach focused on cognitive disorders due to inaccuracy of dementia codes in younger individuals, further analysis is warranted in other older cohorts, and subsequent analyses as this longitudinal cohort ages.

## Conclusions

The results of our study suggest that CUD and TBI are independent risk factors for cognitive disorder and the highest incidence of cognitive disorder is observed in Veterans with comorbid CUD+TBI. TBI and CUD are both independently associated with cognitive impairment. Cognitive impairment is a common post-TBI symptom that may last more than 6 months post-injury (8, 9). Acute inhaled cannabis use is associated with cognitive impairment that may last at least 5 h (38). However, the timing of cognitive disorders is the key point in our study (i.e., time from TBI to ICD codes for dementia diagnosis) and likely indicates permanent cognitive dysfunction after TBI insult. The heterogeneity in impact of CUD on emergence of EOD and Non-EOD subgroup in our cohort, who were relatively young at the time of TBI, may be indicative of the potential harms of cannabis use on long-term cognitive dysfunction. Given that cannabis receptor (CB1R) is enriched in the mesocorticolimbic system (67) and cannabis exposure increases long-term vulnerability to cognitive impairments (68, 69), our results support the long-term harmful effect of cannabis use in patients with cognitive disorder and dementia subtypes that involved brain areas other than frontal and temporal lobes (AD and FTD). Cannabis users showed that the cerebral blood flow reduced in cortical regions and increased in the right precuneus at baseline (70). Also, in the experimental animal's study, noxious effects of chronic cannabis exposure led to higher THC and cannabidiol concentrations in cerebellum and occipital cortex of squirrel monkeys and persisted after discontinuation of the treatment (71). Further studies is needed to evaluate the impact of the chronic cannabis use and structural changes in medial temporal structures and midbrain (40–45). Given the findings of this analysis and the increasing awareness of the potential long-term impacts of combat-related and civilian TBI and the growing rates of CUD, further investigations are warranted.

## Data availability statement

The datasets presented in this article are not readily available because VA regulation required the dataset behind the firewall. Requests to access the datasets should be directed to vinci@va.gov.

## Ethics statement

The studies involving humans were approved by the University of Utah and Stanford University. The studies were conducted in accordance with the local legislation and institutional requirements. Written informed consent for participation was not required from the participants or the participants' legal guardians/next of kin in accordance with the national legislation and institutional requirements.

## Author contributions

AE: Conceptualization, Data curation, Formal analysis, Funding acquisition, Investigation, Methodology, Resources, Software, Validation, Visualization, Writing – original draft, Writing – review & editing. CD-G: Conceptualization, Funding acquisition, Investigation, Methodology, Supervision, Writing – review & editing. TP: Investigation, Methodology, Writing – review & editing. MA: Data curation, Software, Writing – review & editing. CG: Project administration, Resources, Writing – review & editing. AD: Investigation, Writing – review & editing. MM: Project administration, Writing – review & editing. EK: Writing – review & editing, Data curation, Methodology, Validation. DC: Conceptualization, Funding acquisition, Investigation, Supervision, Writing – review & editing. MP: Conceptualization, Funding acquisition, Investigation, Methodology, Supervision, Validation, Writing – review & editing.

## Author disclaimer

Opinions, interpretations, conclusions, and recommendations are those of the author and are not necessarily endorsed by the Department of Defense. Any opinions, findings, conclusions, or recommendations expressed in this publication are those of the author(s) and do not necessarily reflect the views of the U.S. Government, or the U.S. Department of Veterans Affairs, and no official endorsement should be inferred. The views, opinions, and or/findings contained in this abstract are those of the authors and should not be construed as an official Veterans Affairs or Department of Defense position, policy or decision, unless so designated by other official documentation. The content is solely the responsibility of the authors.
