# Supplementary material for: Cannabis use disorder contributes to cognitive dysfunction in Veterans with traumatic brain injury
Source: Front Neurol. 2024 Jan 16;15:1261249. doi: 10.3389/fneur.2024.1261249 (PMC10824930; doi:10.3389/fneur.2024.1261249)
Supplement: Supplementary file 1 [file Data_Sheet_1.pdf]

Supplementary Material

Does Cannabis Use Disorder Contribute to Cognitive Dysfunction in Veterans with TBI?

Table S1. Demographic and Clinical Characteristics of Veterans by CUD and TBI status (N=1,560,556).

	Control N (%)	TBI only N (%)	CUD only N (%)	CUD+TBI N (%)	Total N (%)	SMD TBI	SMD CUD
Overall	1124686 (72.07)	345896 (22.16)	48100 (3.08)	41874 (2.68)	1,560,556 (100)		
Male	902228 (80.22)	304369 (87.99)	39783 (82.71)	38243 (91.33)	1284623 (82.32)	0.222	-0.129
Age at TBI (Mean ± SD)	35.36 (11.60)	33.30 (10.42)	28.43 (8.07)	28.58 (7.29)	34.51 (11.29)	0.210	0.656
Age<65 (at the time of TBI)	1111996 (98.87)	343352 (99.26)	48053 (99.90)	41843 (99.93)	1545244 (99.02)	0.045	0.127
Race/Ethnicity (White)	684525 (60.86)	220561 (63.77)	26501 (55.1)	25796 (61.6)	957383 (61.35)	-0.010	-0.052
Black or African American	232517 (20.67)	54881 (15.87)	13172 (27.38)	7211 (17.22)	307781 (19.72)		
Hispanic or Latino	115010 (10.23)	35771 (10.34)	4353 (9.05)	3629 (8.67)	158763 (10.17)		
Other	86198 (7.66)	33403 (9.66)	3901 (8.11)	5150 (12.3)	128652 (8.24)		
Unknown	6436 (0.57)	1280 (0.37)	173 (0.36)	88 (0.21)	7977 (0.51)		
Education (College and above)	346404 (30.8)	83513 (24.14)	5730 (11.91)	4491 (10.73)	440138 (28.2)	-0.162	-0.448
High School and Less	774755 (68.89)	261783 (75.68)	42282 (87.9)	37330 (89.15)	1116150 (71.52)		
Unknown	3527 (0.31)	600 (0.17)	88 (0.18)	53 (0.13)	4268 (0.27)		
Marital Status (Not married)	544334 (48.4)	162840 (47.08)	33069 (68.75)	27000 (64.48)	767243 (49.16)	-0.006	0.384
Married	579138 (51.49)	182875 (52.87)	15001 (31.19)	14857 (35.48)	791871 (50.74)		
Unknown	1214 (0.11)	181 (0.05)	30 (0.06)	17 (0.04)	1442 (0.09)		
Branch (Air Force)	208515 (18.54)	34084 (9.85)	5661 (11.77)	2487 (5.94)	250747 (16.07)	0.070	0.013
Army	510432 (45.38)	206662 (59.75)	26205 (54.48)	28100 (67.11)	771399 (49.43)		
Marines	161454 (14.36)	61554 (17.8)	6953 (14.46)	6957 (16.61)	236918 (15.18)		
Navy/Coast Guard	243312 (21.63)	43433 (12.56)	9275 (19.28)	4328 (10.34)	300348 (19.25)		
Other	973 (0.09)	163 (0.05)	6 (0.01)	2 (0)	1144 (0.07)		
Rank (Enlisted)	1004715 (89.34)	321134 (92.85)	47300 (98.34)	41262 (98.54)	1414411 (90.64)	0.122	0.343
Officer	105095 (9.34)	20969 (6.06)	672 (1.4)	508 (1.21)	127244 (8.15)		
Warrant	14812 (1.32)	3764 (1.09)	124 (0.26)	102 (0.24)	18802 (1.2)		
Rurality (Rural)	313229 (27.85)	109710 (31.72)	12344 (25.66)	12941 (30.9)	448224 (28.72)	0.086	-0.010

Urban	806537 (71.71)	235011 (67.94)	35677 (74.17)	28840 (68.87)	1106065 (70.88)		
Unknown	4920 (0.44)	1175 (0.34)	79 (0.16)	93 (0.22)	6267 (0.4)		
VA SCD None/0 percent	254515 (22.63)	39619 (11.45)	10384 (21.59)	5060 (12.08)	309578 (19.84)	-0.421	-0.158
10-40 percent	185725 (16.51)	24283 (7.02)	4943 (10.28)	1831 (4.37)	216782 (13.89)		
≥50 percent	684446 (60.86)	281994 (81.53)	32773 (68.14)	34983 (83.54)	1034196 (66.27)		
District (North Atlantic)	246134 (21.89)	71638 (20.71)	9963 (20.71)	8365 (19.98)	336100 (21.54)	-0.034	-0.029
Southeast	229057 (20.37)	67119 (19.4)	10061 (20.92)	7832 (18.7)	314069 (20.13)		
Midwest	217771 (19.36)	70310 (20.33)	9437 (19.62)	8701 (20.78)	306219 (19.62)		
Continental	226839 (20.17)	73873 (21.36)	9917 (20.62)	8982 (21.45)	319611 (20.48)		
Pacific	204855 (18.21)	62948 (18.2)	8720 (18.13)	7994 (19.09)	284517 (18.23)		
Headache	282990 (25.16)	197752 (57.17)	14821 (30.81)	25226 (60.24)	520789 (33.37)	-0.689	-0.245
Other Chronic Pain	884584 (78.65)	314294 (90.86)	41360 (85.99)	39197 (93.61)	1279435 (81.99)	-0.348	-0.229
MAT	29563 (2.63)	19292 (5.58)	10485 (21.8)	11650 (27.82)	70990 (4.55)	-0.198	-0.645
Cancer (based on Oncology visit)	12187 (1.08)	4409 (1.27)	616 (1.28)	631 (1.51)	17843 (1.14)	-0.019	-0.023
SMI	128519 (11.43)	82841 (23.95)	23283 (48.41)	25302 (60.42)	259945 (16.66)	-0.377	-0.919
Depression	389215 (34.61)	190748 (55.15)	36809 (76.53)	35197 (84.05)	651969 (41.78)	-0.450	-0.909
PTSD	292631 (26.02)	225294 (65.13)	28998 (60.29)	36407 (86.94)	583330 (37.38)	-0.876	-0.811
Personality Disorder	22613 (2.01)	15817 (4.57)	8488 (17.65)	10123 (24.17)	57041 (3.66)	-0.192	-0.587
Alcohol Use Disorder	209531 (18.63)	121974 (35.26)	34111 (70.92)	34672 (82.8)	400288 (25.65)	-0.436	-1.280
Opioid Use Disorder	24459 (2.17)	20824 (6.02)	14697 (30.56)	18720 (44.71)	78700 (5.04)	-0.276	-0.939
Other Drug Use Disorder	28869 (2.57)	24150 (6.98)	28052 (58.32)	27556 (65.81)	108627 (6.96)	-0.298	-1.582
Nicotine Use Disorder	178754 (15.89)	93409 (27)	20290 (42.18)	23682 (56.56)	316135 (20.26)	-0.315	-0.678
Anxiety	363430 (32.31)	174680 (50.5)	32448 (67.46)	31843 (76.04)	602401 (38.6)	-0.401	-0.747
Insomnia	207220 (18.42)	117128 (33.86)	10998 (22.86)	16704 (39.89)	352050 (22.56)	-0.366	-0.199
Memory loss	14928 (1.33)	56855 (16.44)	1072 (2.23)	8023 (19.16)	80878 (5.18)	-0.556	-0.200
Congestive Heart Failure	20709 (1.84)	7384 (2.13)	801 (1.67)	864 (2.06)	29758 (1.91)	-0.021	0.004
Peripheral vascular Disease	37248 (3.31)	13281 (3.84)	1046 (2.17)	1245 (2.97)	52820 (3.38)	-0.026	0.052
Cardiac Disease	112527 (10.01)	50198 (14.51)	5921 (12.31)	8178 (19.53)	176824 (11.33)	-0.150	-0.136
Stroke	15412 (1.37)	13779 (3.98)	588 (1.22)	1725 (4.12)	31504 (2.02)	-0.164	-0.039
Diabetes without complication	132115 (11.75)	38745 (11.2)	3509 (7.3)	3163 (7.55)	177532 (11.38)	0.024	0.144
Diabetes with chronic complication	73779 (6.56)	21833 (6.31)	1975 (4.11)	1699 (4.06)	99286 (6.36)	0.016	0.108

Convulsions disorders	91835 (8.17)	87675 (25.35)	11772 (24.47)	20070 (47.93)	211352 (13.54)	-0.505	-0.566
Other Neurologic Disorders (No Convulsions disorders)	5872 (0.52)	7404 (2.14)	380 (0.79)	1131 (2.7)	14787 (0.95)	-0.144	-0.069
Liver Disease	34041 (3.03)	13230 (3.82)	1702 (3.54)	1893 (4.52)	50866 (3.26)	-0.047	-0.042
Chronic Kidney Disease	19286 (1.71)	6535 (1.89)	860 (1.79)	844 (2.02)	27525 (1.76)	-0.014	-0.010
Antidepressants	470233 (41.81)	241002 (69.67)	40135 (83.44)	38477 (91.89)	789847 (50.61)	-0.604	-0.919
Opioids	309917 (27.56)	153330 (44.33)	24297 (50.51)	27719 (66.2)	515263 (33.02)	-0.382	-0.549
Antipsychotics	83881 (7.46)	68233 (19.73)	22497 (46.77)	25526 (60.96)	200137 (12.82)	-0.414	-1.041
Stimulants	49064 (4.36)	31795 (9.19)	4471 (9.3)	5914 (14.12)	91244 (5.85)	-0.201	-0.218
Hypnotics (no Benzodiazepine)	136072 (12.1)	92525 (26.75)	15498 (32.22)	19716 (47.08)	263811 (16.9)	-0.402	-0.549
Benzodiazepine	120202 (10.69)	77849 (22.51)	18676 (38.83)	22139 (52.87)	238866 (15.31)	-0.363	-0.747
Opioid Antagonist	41073 (3.65)	26827 (7.76)	11588 (24.09)	12891 (30.79)	92379 (5.92)	-0.222	-0.649
Anticonvulsants	249851 (22.22)	155541 (44.97)	26564 (55.23)	30831 (73.63)	462787 (29.66)	-0.528	-0.781
Dementia (EOD)	845 (0.08)	527 (0.15)	38 (0.08)	59 (0.14)	1469 (0.09)	-0.023	-0.005

Abbreviations: SCD=Service Connected Disability, TBI= Traumatic Brain Injury, CUD= Cannabis Use Disorder, MAT= Medication-Assisted Treatment, CHF= Congestive Heart Failure, CKD= Chronic Kidney Disease, PTSD= Post Traumatic Stress Disorder, SMI= Severe Mental Illness, DM= Diabetes Mellitus, AD= Alzheimer's disease, FTD=Frontotemporal dementia, EOD= Early Onset Dementia, SMD= Standardized Mean Difference.

Note: The Standardized Mean Difference was, separately calculated to present the potential clinical and structural population differences between the (TBI and Non-TBI) and (CUD and Non-CUD) groups. The statistical difference is significant for all variables (<0.005). The absolute value of standardized mean differences (SMDs) greater than 0.1 were interpreted as an important difference between groups.(Austin, 2009b;Austin, 2009a;Ranganathan et al., 2015)

Table S2. Dementia Incidence Rate (overall and by TBI and CUD status), and Hazard Ratio of Dementia for CUD, TBI and the modifying effect between TBI and CUD.

	Crude	Adjusted1_all
	HR (95% CI)	HR (95% CI)
All types of dementia diagnosis, compared with control group		
	Ref	Ref
TBI only	3.47 (3.33, 3.62)	2.32 (2.13, 2.53)
CUD only	2.31 (2.09, 2.55)	1.79 (1.60, 2.00)
TBI&CUD*	0.77 (0.69, 0.87)	0.78 (0.69, 0.89)
EOD with confirmed disease, compared with control group		
TBI	1.75 (1.54, 1.99)	1.75 (1.30, 2.35)
CUD	0.68 (0.44, 1.07)	1.49 (0.93, 2.39)
TBI&CUD*	1.08 (0.61, 1.91)	1.08 (0.61, 1.92)
All other confirmed dementia (No EOD), compared with control group		
TBI	4.61 (4.32, 4.92)	3.04 (2.68, 3.44)
CUD	2.27 (1.93, 2.68)	1.84 (1.54, 2.20)
TBI&CUD*	0.68 (0.56, 0.82)	0.71 (0.58, 0.86)

Abbreviations: HR= Hazard Ratio, IR= Incidence Rate, CI= Confidence Interval, EOD=Early Onset Dementia, TBI= Traumatic Brain Injury, CUD= Cannabis Use Disorder, PMO= Person Months of Observations, Ref=Reference.

*TBI&CUD: The HR presents the interaction term between TBI and CUD.

Note: The covariates included in the adjusted model: CUD, TBI, sex, age at the time of TBI, TBI severity, race, education, marital status, branch, rank, Rurality, service connected disabilities (percent), District, Headache, Chronic Pain, MAT (recent), Oncology, SMI, Depression, PTSD, Personality Disorder, Alcohol Use Disorder, Opioid Use Disorder, Other SUD, Nicotine Use disorder, anxiety, insomnia, CHF, Perivascular disease, Cardiac disease, Stroke, DM, DM with complications, convulsions disorders, Neurologic disorder (No Convulsions disorders), Liver Disease, CKD, and death.

Table S3: Variables Included in our study.

**Variables**	**Description**	ICD-9/10 codes
**Outcomes (dementia and CUD)**	Dementia types included in the study: Creutzfeldt-Jakob Disease, Unspecified; Variant Creutzfeldt-Jakob Disease; Other Creutzfeldt-Jakob Disease; Progressive Multifocal Leukoencephalopathy; Gerstmann-Straussler-Scheinker Syndrome; Other Atypical Virus Infections Of Central Nervous System; Atypical Virus Infection Of Central Nervous System, Unspecified; Vascular Dementia Without Behavioral Disturbance; Vascular Dementia With Behavioral Disturbance; Dementia In Other Diseases Classified Elsewhere Without Behavioral Disturbance; Dementia In Other Diseases Classified Elsewhere With Behavioral Disturbance; Unspecified Dementia Without Behavioral Disturbance; Unspecified Dementia With Behavioral Disturbance; Alcohol Dependence With Alcohol-Induced Persisting Dementia; Alcohol Use, Unspecified With Alcohol-Induced Persisting Dementia; Sedative, Hypnotic Or Anxiolytic Dependence With Sedative, Hypnotic Or Anxiolytic-Induced Persisting Dementia; Sedative, Hypnotic Or Anxiolytic Use, Unspecified With Sedative, Hypnotic Or Anxiolytic-Induced Persisting Dementia; Inhalant Abuse With Inhalant-Induced Dementia; Inhalant Dependence With Inhalant-Induced Dementia; Inhalant Use, Unspecified With Inhalant-Induced Persisting Dementia; Other Psychoactive Substance Abuse With Psychoactive Substance-Induced Persisting Dementia; Other Psychoactive Substance Dependence With Psychoactive Substance-Induced Persisting Dementia; Other Psychoactive Substance Use, Unspecified With Psychoactive Substance-Induced Persisting Dementia; Progressive Supranuclear Ophthalmoplegia [Steele-Richardson-Olszewski]; Alzheimer's	046.11', '046.19', '046.3', '046.71', '046.79', '046.9', '290.0', '290.10', '290.11', '290.12', '290.13', '290.20', '290.21', '290.3', '290.40', '290.41', '290.42', '290.43', '291.1', '291.2', '292.82', '294.1', '294.10', '294.11', '294.20', '294.21', '331.0', '331.11', '331.19', '331.82', 'A81.00', 'A81.01', 'A81.09', 'A81.2', 'A81.82', 'A81.89', 'A81.9', 'F01.50', 'F01.51', 'F02.80', 'F02.81', 'F03.90', 'F03.91', 'F10.27', 'F10.97', 'F13.27', 'F13.97', 'F18.17', 'F18.27', 'F18.97', 'F19.17', 'F19.27', 'F19.97', 'G23.1', 'G30.0', 'G30.1', 'G30.8', 'G30.9', 'G31.01', 'G31.09', 'G31.83', 'G90.3',

	Disease With Early Onset; Alzheimer's Disease With Late Onset; Other Alzheimer's Disease; Alzheimer's Disease, Unspecified; Pick's Disease; Other Frontotemporal Dementia; Dementia With Lewy Bodies; Multi-System Degeneration Of The Autonomic Nervous System; Dementia In Other Diseases Classified Elsewhere Without Behavioral Disturbance/Human; Immunodeficiency Virus [HIV] Disease; Dementia In Other Diseases Classified Elsewhere With Behavioral Disturbance/HIV Disease; Dementia In Other Diseases Classified Elsewhere Without Behavioral Disturbance/Huntington's Disease; Dementia In Other Diseases Classified Elsewhere With Behavioral Disturbance/Huntington's Disease; Dementia In Other Diseases Classified Elsewhere Without Behavioral Disturbance/Parkinson's Disease; Dementia In Other Diseases Classified Elsewhere With Behavioral Disturbance/Parkinson's Disease; Dementia In Other Diseases Classified Elsewhere Without Behavioral Disturbance/Idiopathic Normal Pressure Hydrocephalus; Dementia In Other Diseases Classified Elsewhere With Behavioral Disturbance/Idiopathic Normal Pressure Hydrocephalus (SOURCE: Chief, Dementia Initiatives, VHA Geriatrics and Extended Care).	
	**Note:** A diagnosis of dementia was identified using ICD-9/10 codes after TBI index date. The ICD codes provided by Veterans Health Administration (VHA) geriatrics and extended care. To address a previously identified limitation of ICD codes not accurately capturing dementia in patients under the age of 65 (Salem et al., 2014), we expanded the dementia diagnosis definition in our cohort to also include cognitive disorder.	

	CUD: ICD-9 codes= Cannabis dependence (304.3), and Nondependent cannabis abuse (305.2), and ICD-10 codes= Cannabis-related disorders (F12).	304.3, 305.2, F12 (F12.1, F12.2, and F12.9)
**Comorbid Conditions after TBI index date, using a conceptual framework (Nuckols et al., 2013)**	Demographic and military characteristics:	
	Verified by LIMBIC-CENC: sex, Age at the time of TBI, TBI severity, branch, rank, Rurality, service-connected disability groupings	
	Using VA Corporate Data Warehouse: Race, Education, Marital status, District, Death	
	Clinical and mental health characteristics	
	Selected Elixhauser co-morbidities were identified as categorical variables (yes/no) based on ICD-9 or ICD-10 codes identified as: congestive heart failure, cardiac arrhythmias, peripheral vascular disorders, uncomplicated diabetes, complicated diabetes, depression, liver disease, Chronic Kidney Disease, alcohol abuse, drug abuse, and insomnia, sleep Apnea (Elixhauser et al., 1998).	
	Post-traumatic stress disorder (PTSD), anxiety, (Kennedy et al., 2022).	('309.81', 'F43.10', 'F43.11', 'F43.12')
	Headache, Stroke, Convulsions disorders, Neurologic disorder (Other than convulsions disorders) (Hai et al., 2023),	

	Chronic Pain (other than headache): including Menstrual Disorders, Vulvodynia, Interstitial Cystitis, Irritable Bowel Syndrome, Crystal Arthropathies, Back pain, Osteoarthritis, Myalgia/Myositis, Arthropathy/Tendinopathy, Neck/Spine pain, Neuropathy, Spinal Cord Injury, Endometriosis.	('739.9', '789', '789.0', '789.00', '789.01', '789.02', '789.03', '789.04', '789.05', '789.06', '789.07', '789.09', 'G43.D0', 'G43.D1', 'M99.09', 'R10.10', 'R10.11', 'R10.12', 'R10.13', 'R10.30', 'R10.31', 'R10.32', 'R10.33', 'R10.84', 'R10.9', '564.1', 'K58.0', 'K58.1', 'K58.2', 'K58.8', 'K58.9')('349.39', '353.1', '353.3', '353.4', '720.0', '720.1', '720.2', '720.81', '720.89', '720.9', '721.2', '721.3', '721.41', '721.42', '721.5', '721.6', '721.7', '721.8', '721.9', '721.90', '721.91', '722', '722.1', '722.10', '722.11', '722.2', '722.3', '722.30', '722.31', '722.32', '722.39', '722.5', '722.51', '722.52', '722.6', '722.7', '722.70', '722.72', '722.73', '722.8', '722.80', '722.82', '722.83', '722.9', '722.90', '722.92', '722.93', '724.0', '724.00', '724.01', '724.02', '724.03', '724.09', '724.1', '724.2', '724.3', '724.4', '724.5', '724.6', '724.7', '724.70', '724.71', '724.79', '724.8', '724.9', '738.3', '738.4', '738.5', '738.6', '739.2', '739.3', '739.4', '739.5', '756.11', '756.12', '756.13', '756.15', '756.16', '756.17', '756.19', '839.2', '839.20', '839.21', '839.40', '839.41', '839.42', '839.49', '846.0', '846.1', '846.2', '846.3', '846.8', '846.9', '847.1', '847.2', '847.3', '847.4', '847.9', '848.5', 'G54.1', 'G54.3', 'G54.4', 'G96.11', 'L40.53', 'M08.1', 'M25.78', 'M43.00', 'M43.04', 'M43.05', 'M43.06', 'M43.07', 'M43.08', 'M43.09', 'M43.10', 'M43.14', 'M43.15', 'M43.16', 'M43.17', 'M43.18', 'M43.19', 'M43.5X4', 'M43.5X5', 'M43.5X6', 'M43.5X7', 'M43.5X8', 'M43.5X9', 'M43.8X4', 'M43.8X5', 'M43.8X6', 'M43.8X7', 'M43.8X8', 'M43.8X9', 'M43.9', 'M45.0', 'M45.4', 'M45.5', 'M45.6', 'M45.7', 'M45.8', 'M45.9', 'M46.00', 'M46.04', 'M46.05', 'M46.06', 'M46.07', 'M46.08', 'M46.09', 'M46.1', 'M46.40', 'M46.44', 'M46.45', 'M46.46', 'M46.47', 'M46.48', 'M46.49', 'M46.80', 'M46.84', 'M46.85', 'M46.86', 'M46.87', 'M46.88', 'M46.89', 'M46.90', 'M46.94', 'M46.95', 'M46.96', 'M46.97', 'M46.98', 'M46.99', 'M47.10', 'M47.14', 'M47.15', 'M47.16', 'M47.20', 'M47.24', 'M47.25', 'M47.26', 'M47.27', 'M47.28', 'M47.814',

		'M47.815', 'M47.816', 'M47.817', 'M47.818', 'M47.819', 'M47.894', 'M47.895', 'M47.896', 'M47.897', 'M47.898', 'M47.899', 'M47.9', 'M48.00', 'M48.04', 'M48.05', 'M48.06', 'M48.07', 'M48.08', 'M48.10', 'M48.14', 'M48.15', 'M48.16', 'M48.17', 'M48.18', 'M48.19', 'M48.20', 'M48.24', 'M48.25', 'M48.26', 'M48.27', 'M48.30', 'M48.34', 'M48.35', 'M48.36', 'M48.37', 'M48.38', 'M48.8X4', 'M48.8X5', 'M48.8X6', 'M48.8X7', 'M48.8X8', 'M48.8X9', 'M48.9', 'M49.80', 'M49.84', 'M49.85', 'M49.86', 'M49.87', 'M49.88', 'M49.89', 'M51.04', 'M51.05', 'M51.06', 'M51.14', 'M51.15', 'M51.16', 'M51.17', 'M51.24', 'M51.25', 'M51.26', 'M51.27', 'M51.34', 'M51.35', 'M51.36', 'M51.37', 'M51.44', 'M51.45', 'M51.46', 'M51.47', 'M51.84', 'M51.85', 'M51.86', 'M51.87', 'M51.9', 'M53.2X4', 'M53.2X5', 'M53.2X6', 'M53.2X7', 'M53.2X8', 'M53.2X9', 'M53.3', 'M53.80', 'M53.84', 'M53.85', 'M53.86', 'M53.87', 'M53.88', 'M53.9', 'M54.00', 'M54.04', 'M54.05', 'M54.06', 'M54.07', 'M54.08', 'M54.09', 'M54.10', 'M54.14', 'M54.15', 'M54.16', 'M54.17', 'M54.18', 'M54.30', 'M54.31', 'M54.32', 'M54.40', 'M54.41', 'M54.42', 'M54.5', 'M54.6', 'M54.89', 'M54.9', 'M62.830', 'M95.4', 'M95.5', 'M96.1', 'M99.02', 'M99.03', 'M99.04', 'M99.05', 'M99.12', 'M99.14', 'M99.18', 'M99.53', 'M99.73', 'M99.79', 'M99.82', 'M99.83', 'M99.84', 'M99.85', 'M99.88', 'Q67.5', 'Q76.0', 'Q76.1', 'Q76.2', 'Q76.3', 'Q76.414', 'Q76.415', 'Q76.419', 'Q76.49', 'S23.100A', 'S23.101A', 'S23.101D', 'S23.101S', 'S23.111A', 'S23.111D', 'S23.111S', 'S23.121A', 'S23.121D', 'S23.121S', 'S23.123A', 'S23.123D', 'S23.123S', 'S23.131A', 'S23.131D', 'S23.131S', 'S23.133A', 'S23.133D', 'S23.133S', 'S23.141A', 'S23.141D', 'S23.141S', 'S23.143A', 'S23.143D', 'S23.143S', 'S23.151A', 'S23.151D', 'S23.151S', 'S23.153A', 'S23.153D', 'S23.153S', 'S23.161A', 'S23.161D', 'S23.161S', 'S23.163A', 'S23.163D', 'S23.163S', 'S23.171A', 'S23.171D', 'S23.171S', 'S23.20XA', 'S23.20XD',

		'S23.20XS', 'S23.29XA', 'S23.29XD', 'S23.29XS', 'S23.3XXA', 'S23.3XXD', 'S23.3XXS', 'S23.8XXA', 'S23.8XXD', 'S23.8XXS', 'S23.9XXA', 'S23.9XXD', 'S23.9XXS', 'S29.019A', 'S29.019D', 'S29.019S', 'S33.0XXA', 'S33.100A', 'S33.101A', 'S33.101D', 'S33.101S', 'S33.111A', 'S33.111D', 'S33.111S', 'S33.121A', 'S33.121D', 'S33.121S', 'S33.131A', 'S33.131D', 'S33.131S', 'S33.140A', 'S33.140D', 'S33.141A', 'S33.141D', 'S33.141S', 'S33.2XXA', 'S33.2XXD', 'S33.2XXS', 'S33.30XA', 'S33.30XD', 'S33.30XS', 'S33.39XA', 'S33.39XD', 'S33.39XS', 'S33.5XXA', 'S33.5XXD', 'S33.5XXS', 'S33.6XXA', 'S33.6XXD', 'S33.6XXS', 'S33.8XXA', 'S33.8XXD', 'S33.8XXS', 'S33.9XXA', 'S33.9XXD', 'S33.9XXS', 'S39.012A', 'S39.012D', 'S39.012S', 'S39.92XA')('729.1', 'D86.87', 'M60.80', 'M60.811', 'M60.812', 'M60.819', 'M60.821', 'M60.822', 'M60.829', 'M60.831', 'M60.851', 'M60.852', 'M60.859', 'M60.861', 'M60.862', 'M60.869', 'M60.871', 'M60.872', 'M60.879', 'M60.89', 'M60.9', 'M79.1', 'M79.7') ('307.81', '339', '339.0', '339.00', '339.01', '339.02', '339.03', '339.04', '339.05', '339.09', '339.1', '339.10', '339.11', '339.12', '339.2', '339.20', '339.21', '339.22', '339.4', '339.41', '339.42', '339.43', '339.44', '339.8', '339.81', '339.82', '339.83', '339.84', '339.85', '339.89', '346', '346.0', '346.00', '346.01', '346.02', '346.03', '346.1', '346.10', '346.11', '346.12', '346.13', '346.2', '346.20', '346.21', '346.22', '346.23', '346.3', '346.30', '346.31', '346.32', '346.33', '346.4', '346.40', '346.41', '346.42', '346.43', '346.5', '346.50', '346.51', '346.52', '346.53', '346.6', '346.60', '346.61', '346.62', '346.63', '346.7', '346.70', '346.71', '346.72', '346.73', '346.8', '346.80', '346.81', '346.82', '346.83', '346.9', '346.90', '346.91', '346.92', '346.93', '784.0', 'G43.001', 'G43.009', 'G43.011', 'G43.019', 'G43.101', 'G43.109', 'G43.109A', 'G43.111', 'G43.119', 'G43.401', 'G43.409', 'G43.411', 'G43.419', 'G43.501', 'G43.509',

		'G43.511', 'G43.519', 'G43.601', 'G43.609', 'G43.611', 'G43.619', 'G43.701', 'G43.709', 'G43.711', 'G43.719', 'G43.801', 'G43.809', 'G43.811', 'G43.819', 'G43.821', 'G43.829', 'G43.831', 'G43.839', 'G43.901', 'G43.909', 'G43.911', 'G43.919', 'G43.B0', 'G43.B1', 'G43.C0', 'G43.C1', 'G44.001', 'G44.009', 'G44.011', 'G44.019', 'G44.021', 'G44.029', 'G44.039', 'G44.049', 'G44.051', 'G44.059', 'G44.099', 'G44.1', 'G44.201', 'G44.209', 'G44.211', 'G44.219', 'G44.221', 'G44.229', 'G44.301', 'G44.309', 'G44.311', 'G44.319', 'G44.321', 'G44.329', 'G44.51', 'G44.52', 'G44.53', 'G44.59', 'G44.81', 'G44.82', 'G44.83', 'G44.84', 'G44.85', 'G44.89', 'M54.81', 'R51') ('711', '716', '716.1', '716.10', '716.11', '716.12', '716.13', '716.14', '716.15', '716.16', '716.17', '716.18', '716.19', '716.4', '716.40', '716.41', '716.42', '716.43', '716.44', '716.45', '716.46', '716.47', '716.48', '716.49', '716.5', '716.50', '716.51', '716.52', '716.53', '716.54','716.55', '716.56', '716.57', '716.58', '716.59', '716.6', '716.60', '716.61', '716.62', '716.63', '716.64', '716.65', '716.66', '716.67', '716.68', '716.8', '716.80', '716.81', '716.82', '716.83', '716.84', '716.85', '716.86', '716.87', '716.88', '716.89', '716.9', '716.90', '716.91', '716.92', '716.93', '716.94', '716.95', '716.96', '716.97', '716.98', '716.99', '717.81', '717.82', '717.83', '717.84', '717.85', '718.0', '718.00', '718.01', '718.02', '718.03', '718.04', '718.05', '718.07', '718.08', '718.09', '718.3', '718.30', '718.31', '718.32', '718.33', '718.34', '718.35', '718.36', '718.37', '718.38', '718.39', '718.4', '718.40', '718.41', '718.42', '718.43', '718.44', '718.45', '718.46', '718.47', '718.48', '718.49', '718.5', '718.50', '718.51', '718.52', '718.53', '718.54', '718.55', '718.56', '718.57', '718.58', '718.59', '719', '719.4', '719.40', '719.41', '719.42', '719.43', '719.44', '719.45', '719.46', '719.47', '719.48', '719.49', '726', '726.0', '726.10', '726.11', '726.12', '726.19', '726.2', '726.30', '726.31', '726.32', '726.33', '726.39',

		'726.4', '726.5', '726.6', '726.60', '726.61', '726.62', '726.64', '726.65', '726.69', '726.70', '726.71', '726.72', '726.79', '726.90', '726.91', '727.2', '727.3', '727.40', '727.49', '727.51', '727.61', '727.82', '727.83', '727.89', '727.9', '728.4', 'M06.219', 'M12.50', 'M12.511', 'M12.512', 'M12.519', 'M12.521', 'M12.522', 'M12.529', 'M12.531', 'M12.532', 'M12.539', 'M12.541', 'M12.542', 'M12.549', 'M12.551', 'M12.552', 'M12.559', 'M12.561', 'M12.562', 'M12.569', 'M12.571', 'M12.572', 'M12.579', 'M12.58', 'M12.59', 'M12.80', 'M12.811', 'M12.812', 'M12.819', 'M12.821', 'M12.822', 'M12.829', 'M12.831', 'M12.832', 'M12.839', 'M12.841', 'M12.842', 'M12.849', 'M12.851', 'M12.852', 'M12.859', 'M12.861', 'M12.862', 'M12.869', 'M12.871', 'M12.872', 'M12.879', 'M12.88', 'M12.89', 'M12.9', 'M13.0', 'M13.10', 'M13.111', 'M13.112', 'M13.119', 'M13.121', 'M13.122', 'M13.129', 'M13.131', 'M13.132', 'M13.139', 'M13.141', 'M13.142', 'M13.149', 'M13.151', 'M13.152', 'M13.159', 'M13.161', 'M13.162', 'M13.169', 'M13.171', 'M13.172', 'M13.179', 'M22.01', 'M22.02', 'M22.10', 'M22.11', 'M22.12', 'M23.50', 'M23.51', 'M23.52', 'M24.10', 'M24.111', 'M24.112', 'M24.119', 'M24.121', 'M24.122', 'M24.129', 'M24.131', 'M24.132', 'M24.139', 'M24.141', 'M24.142', 'M24.149', 'M24.151', 'M24.152', 'M24.159', 'M24.171', 'M24.172', 'M24.173', 'M24.174', 'M24.175', 'M24.176', 'M24.20', 'M24.211', 'M24.212', 'M24.219', 'M24.221', 'M24.222', 'M24.229', 'M24.231', 'M24.232', 'M24.239', 'M24.241', 'M24.242', 'M24.249', 'M24.251', 'M24.252', 'M24.259', 'M24.271', 'M24.272', 'M24.273', 'M24.274', 'M24.275', 'M24.276', 'M24.28', 'M24.40', 'M24.411', 'M24.412', 'M24.419', 'M24.421', 'M24.422', 'M24.429', 'M24.431', 'M24.432', 'M24.439', 'M24.441', 'M24.442', 'M24.443', 'M24.444', 'M24.445', 'M24.446', 'M24.451', 'M24.452', 'M24.459', 'M24.461', 'M24.462',

		'M24.469', 'M24.471', 'M24.472', 'M24.473', 'M24.474', 'M24.475', 'M24.476', 'M24.477', 'M24.478', 'M24.479', 'M24.50', 'M24.511', 'M24.512', 'M24.519', 'M24.521', 'M24.522', 'M24.529', 'M24.531', 'M24.532', 'M24.539', 'M24.541', 'M24.542', 'M24.549', 'M24.551', 'M24.552', 'M24.559', 'M24.561', 'M24.562', 'M24.569', 'M24.571', 'M24.572', 'M24.573', 'M24.574', 'M24.575', 'M24.576', 'M24.60', 'M24.611', 'M24.612', 'M24.619', 'M24.621', 'M24.622', 'M24.629', 'M24.631', 'M24.632', 'M24.639', 'M24.641', 'M24.642', 'M24.649', 'M24.651', 'M24.652', 'M24.659', 'M24.661', 'M24.662', 'M24.669', 'M24.671', 'M24.672', 'M24.673', 'M24.674', 'M24.675', 'M24.676', 'M25.50', 'M25.511', 'M25.512', 'M25.519', 'M25.521', 'M25.522', 'M25.529', 'M25.531', 'M25.532', 'M25.539', 'M25.541', 'M25.542', 'M25.549', 'M25.551', 'M25.552', 'M25.559', 'M25.561', 'M25.562', 'M25.569', 'M25.571', 'M25.572', 'M25.579', 'M25.70', 'M25.711', 'M25.712', 'M25.719', 'M25.721', 'M25.722', 'M25.729', 'M25.731', 'M25.732', 'M25.739', 'M25.741', 'M25.742', 'M25.749', 'M25.751', 'M25.752', 'M25.759', 'M25.761', 'M25.762', 'M25.769', 'M25.771', 'M25.772', 'M25.773', 'M25.774', 'M25.775', 'M25.776', 'M62.411', 'M62.412', 'M62.419', 'M65.011', 'M65.012', 'M65.019', 'M65.10', 'M65.811', 'M65.812', 'M65.819', 'M66.211', 'M66.212', 'M66.219', 'M66.311', 'M66.312', 'M66.319', 'M66.811', 'M66.812', 'M66.819', 'M67.00', 'M67.01', 'M67.02', 'M67.50', 'M67.51', 'M67.52', 'M67.80', 'M67.811', 'M67.812', 'M67.813', 'M67.814', 'M67.819', 'M67.821', 'M67.822', 'M67.823', 'M67.824', 'M67.829', 'M67.831', 'M67.832', 'M67.833', 'M67.834', 'M67.839', 'M67.841', 'M67.842', 'M67.843', 'M67.844', 'M67.849', 'M67.851', 'M67.852', 'M67.853', 'M67.854', 'M67.859', 'M67.861', 'M67.862', 'M67.863', 'M67.864', 'M67.869', 'M67.871',

		'M67.872', 'M67.873', 'M67.874', 'M67.879', 'M67.88', 'M67.89', 'M67.90', 'M67.911', 'M67.912', 'M67.919', 'M67.921', 'M67.922', 'M67.929', 'M67.931', 'M67.932', 'M67.939', 'M67.941', 'M67.942', 'M67.949', 'M67.951', 'M67.952', 'M67.959', 'M67.961', 'M67.962', 'M67.969', 'M67.971', 'M67.972', 'M67.979', 'M67.98', 'M67.99', 'M70.031', 'M70.032', 'M70.039', 'M70.10', 'M70.11', 'M70.12', 'M70.20', 'M70.21', 'M70.22', 'M70.30', 'M70.31', 'M70.32', 'M70.40', 'M70.41', 'M70.42', 'M70.50', 'M70.51', 'M70.52', 'M70.60', 'M70.61', 'M70.62', 'M70.70', 'M70.71', 'M70.72', 'M70.90', 'M71.00', 'M71.011', 'M71.012', 'M71.019', 'M71.021', 'M71.022', 'M71.029', 'M71.031', 'M71.032', 'M71.039', 'M71.041', 'M71.042', 'M71.049', 'M71.051', 'M71.052', 'M71.059', 'M71.061', 'M71.062', 'M71.069', 'M71.071', 'M71.072', 'M71.079', 'M71.08', 'M71.09', 'M71.10', 'M71.121', 'M71.122', 'M71.129', 'M71.161', 'M71.162', 'M71.20', 'M71.21', 'M71.22', 'M71.30', 'M71.311', 'M71.312', 'M71.319', 'M71.321', 'M71.322', 'M71.329', 'M71.331', 'M71.332', 'M71.339', 'M71.341', 'M71.342', 'M71.349', 'M71.351', 'M71.352', 'M71.359', 'M71.371', 'M71.372', 'M71.379', 'M71.38', 'M71.39', 'M71.40', 'M71.421', 'M71.422', 'M71.429', 'M71.431', 'M71.432', 'M71.439', 'M71.441', 'M71.442', 'M71.449', 'M71.451', 'M71.452', 'M71.459', 'M71.461', 'M71.462', 'M71.469', 'M71.471', 'M71.472', 'M71.479', 'M71.48', 'M71.49', 'M71.50', 'M71.521', 'M71.522', 'M71.529', 'M71.531', 'M71.532', 'M71.539', 'M71.541', 'M71.542', 'M71.549', 'M71.551', 'M71.552', 'M71.559', 'M71.561', 'M71.562', 'M71.569', 'M71.571', 'M71.572', 'M71.579', 'M71.58', 'M71.80', 'M71.811', 'M71.812', 'M71.819', 'M71.821', 'M71.822', 'M71.829', 'M71.831', 'M71.832', 'M71.839', 'M71.841', 'M71.842', 'M71.849', 'M71.851', 'M71.852', 'M71.859', 'M71.861',

		'M71.862', 'M71.869', 'M71.871', 'M71.872', 'M71.879', 'M71.88', 'M71.89', 'M71.9', 'M75.00', 'M75.01', 'M75.02', 'M75.100', 'M75.101', 'M75.102', 'M75.110', 'M75.111', 'M75.112', 'M75.120', 'M75.121', 'M75.122', 'M75.20', 'M75.21', 'M75.22', 'M75.30', 'M75.31', 'M75.32', 'M75.40', 'M75.41', 'M75.42', 'M75.50', 'M75.51', 'M75.52', 'M75.80', 'M75.81', 'M75.82', 'M76.00', 'M76.02', 'M76.10', 'M76.11', 'M76.12', 'M76.20', 'M76.21', 'M76.22', 'M76.40', 'M76.41', 'M76.42', 'M76.50', 'M76.51', 'M76.52', 'M76.60', 'M76.61', 'M76.62', 'M76.811', 'M76.812', 'M76.819', 'M76.821', 'M76.822', 'M76.829', 'M76.891', 'M76.892', 'M76.899', 'M77.00', 'M77.01', 'M77.02', 'M77.10', 'M77.11', 'M77.12', 'M77.20', 'M77.21', 'M77.22', 'M77.9', 'M94.8X1') ('715', '715.0', '715.00', '715.04', '715.09', '715.1', '715.10', '715.11', '715.12', '715.13', '715.14', '715.15', '715.16', '715.17', '715.18', '715.2', '715.20', '715.21', '715.22', '715.23', '715.24', '715.25', '715.26', '715.27', '715.28', '715.3', '715.30', '715.31', '715.32', '715.33', '715.34', '715.35', '715.36', '715.37', '715.38', '715.8', '715.80', '715.89', '715.9', '715.90', '715.91', '715.92', '715.93', '715.94', '715.95', '715.96', '715.97', '715.98', 'M15.0', 'M15.1', 'M15.2', 'M15.3', 'M15.4', 'M15.8', 'M15.9', 'M16.0', 'M16.10', 'M16.11', 'M16.12', 'M16.2', 'M16.30', 'M16.31', 'M16.32', 'M16.4', 'M16.50', 'M16.51', 'M16.52', 'M16.6', 'M16.7', 'M16.9', 'M17.0', 'M17.10', 'M17.11', 'M17.12', 'M17.2', 'M17.30', 'M17.31', 'M17.32', 'M17.4', 'M17.5', 'M17.9', 'M18.0', 'M18.10', 'M18.11', 'M18.12', 'M18.2', 'M18.30', 'M18.31', 'M18.32', 'M18.4', 'M18.50', 'M18.51', 'M18.52', 'M18.9', 'M19.011', 'M19.012', 'M19.019', 'M19.021', 'M19.022', 'M19.029', 'M19.031', 'M19.032', 'M19.039', 'M19.041', 'M19.042', 'M19.049', 'M19.071', 'M19.072', 'M19.079', 'M19.111', 'M19.112', 'M19.119', 'M19.121', 'M19.122', 'M19.129', 'M19.131', 'M19.132', 'M19.139', 'M19.141', 'M19.142', 'M19.149',

		'M19.171', 'M19.172', 'M19.179', 'M19.211', 'M19.212', 'M19.219', 'M19.221', 'M19.222', 'M19.229', 'M19.231', 'M19.232', 'M19.239', 'M19.241', 'M19.242', 'M19.249', 'M19.271', 'M19.272', 'M19.279', 'M19.90', 'M19.91', 'M19.92', 'M19.93', 'M24.7')('274', '274.0', '274.00', '274.01', '274.02', '274.03', '274.10', '274.19', '274.81', '274.82', '274.89', '274.9', '712', '712.1', '712.10', '712.11', '712.12', '712.13', '712.14', '712.15', '712.16', '712.17', '712.18', '712.19', '712.2', '712.20', '712.21', '712.22', '712.23', '712.24', '712.25', '712.26', '712.27', '712.28', '712.29', '712.3', '712.30', '712.31', '712.32', '712.33', '712.34', '712.35', '712.36', '712.37', '712.38', '712.39', '712.8', '712.80', '712.81', '712.82', '712.83', '712.84', '712.85', '712.86', '712.87', '712.88', '712.89', '712.9', '712.90', '712.91', '712.92', '712.93', '712.94', '712.95', '712.96', '712.97', '712.98', '712.99', '717.7', 'M10.00', 'M10.011', 'M10.012', 'M10.019', 'M10.021', 'M10.022', 'M10.029', 'M10.031', 'M10.032', 'M10.039', 'M10.041', 'M10.042', 'M10.049', 'M10.051', 'M10.052', 'M10.059', 'M10.061', 'M10.062', 'M10.069', 'M10.071', 'M10.072', 'M10.079', 'M10.08', 'M10.09', 'M10.10', 'M10.172', 'M10.179', 'M10.20', 'M10.221', 'M10.241', 'M10.242', 'M10.261', 'M10.262', 'M10.269', 'M10.271', 'M10.272', 'M10.279', 'M10.30', 'M10.311', 'M10.312', 'M10.319', 'M10.321', 'M10.322', 'M10.329', 'M10.331', 'M10.332', 'M10.339', 'M10.341', 'M10.342', 'M10.349', 'M10.351', 'M10.352', 'M10.359', 'M10.361', 'M10.362', 'M10.369', 'M10.371', 'M10.372', 'M10.379', 'M10.38', 'M10.39', 'M10.40', 'M10.411', 'M10.412', 'M10.419', 'M10.421', 'M10.422', 'M10.429', 'M10.431', 'M10.432', 'M10.439', 'M10.441', 'M10.442', 'M10.449', 'M10.451', 'M10.452', 'M10.459', 'M10.461', 'M10.462', 'M10.469', 'M10.471', 'M10.472', 'M10.479', 'M10.48', 'M10.49', 'M10.9', 'M11.162', 'M11.20', 'M11.211', 'M11.212',

		'M11.219', 'M11.221', 'M11.222', 'M11.229', 'M11.231', 'M11.232', 'M11.239', 'M11.241', 'M11.242', 'M11.249', 'M11.251', 'M11.252', 'M11.259', 'M11.261', 'M11.262', 'M11.269', 'M11.271', 'M11.272', 'M11.279', 'M11.28', 'M11.29', 'M11.80', 'M11.811', 'M11.812', 'M11.819', 'M11.821', 'M11.822', 'M11.829', 'M11.831', 'M11.832', 'M11.839', 'M11.841', 'M11.842', 'M11.849', 'M11.851', 'M11.852', 'M11.859', 'M11.861', 'M11.862', 'M11.869', 'M11.871', 'M11.872', 'M11.879', 'M11.88', 'M11.89', 'M11.9', 'M1A.00X0', 'M1A.00X1', 'M1A.0110', 'M1A.0111', 'M1A.0120', 'M1A.0121', 'M1A.0190', 'M1A.0191', 'M1A.0210', 'M1A.0211', 'M1A.0220', 'M1A.0221', 'M1A.0290', 'M1A.0291', 'M1A.0310', 'M1A.0311', 'M1A.0320', 'M1A.0321', 'M1A.0390', 'M1A.0391', 'M1A.0410', 'M1A.0411', 'M1A.0420', 'M1A.0421', 'M1A.0490', 'M1A.0491', 'M1A.0510', 'M1A.0511', 'M1A.0520', 'M1A.0521', 'M1A.0590', 'M1A.0591', 'M1A.0610', 'M1A.0611', 'M1A.0620', 'M1A.0621', 'M1A.0690', 'M1A.0691', 'M1A.0710', 'M1A.0711', 'M1A.0720', 'M1A.0721', 'M1A.0790', 'M1A.0791', 'M1A.08X0', 'M1A.08X1', 'M1A.09X0', 'M1A.09X1', 'M1A.10X0', 'M1A.1421', 'M1A.1690', 'M1A.1720', 'M1A.1790', 'M1A.20X0', 'M1A.2211', 'M1A.2720', 'M1A.29X0', 'M1A.30X0', 'M1A.30X1', 'M1A.3320', 'M1A.3421', 'M1A.3490', 'M1A.3491', 'M1A.3710', 'M1A.3711', 'M1A.3720', 'M1A.3721', 'M1A.3790', 'M1A.39X0', 'M1A.39X1', 'M1A.40X0', 'M1A.40X1', 'M1A.4490', 'M1A.4710', 'M1A.4720', 'M1A.4791', 'M1A.49X0', 'M1A.49X1', 'M1A.9XX0', 'M1A.9XX1', 'M22.40', 'M22.41', 'M22.42', 'M94.261', 'M94.262', 'M94.269') ('353.2', '721.0', '721.1', '722.0', '722.4', '722.71', '722.81', '722.91', '723.0', '723.1', '723.2', '723.3', '723.4', '723.5', '723.6', '723.7', '723.8', '723.9', '738.2', '739.1',

		'839.0', '839.00', '839.01', '839.02', '839.03', '839.04', '839.05', '839.06', '839.07', '839.08', '847.0', '848.2', 'G54.2', 'M43.01', 'M43.02', 'M43.03', 'M43.11', 'M43.12', 'M43.13', 'M43.3', 'M43.4', 'M43.5X2', 'M43.5X3', 'M43.6', 'M43.8X1', 'M43.8X2', 'M43.8X3', 'M45.1', 'M45.2', 'M45.3', 'M46.01', 'M46.02', 'M46.03', 'M46.41', 'M46.42', 'M46.43', 'M46.81', 'M46.82', 'M46.83', 'M46.91', 'M46.92', 'M46.93', 'M47.11', 'M47.12', 'M47.13', 'M47.22', 'M47.811', 'M47.812', 'M47.813', 'M47.891', 'M47.892', 'M47.893', 'M48.01', 'M48.02', 'M48.03', 'M48.11', 'M48.12', 'M48.13', 'M48.21', 'M48.22', 'M48.23', 'M48.31', 'M48.32', 'M48.33', 'M48.8X1', 'M48.8X2', 'M48.8X3', 'M49.81', 'M49.82', 'M49.83', 'M50.00', 'M50.01', 'M50.020', 'M50.021', 'M50.022', 'M50.023', 'M50.03', 'M50.10', 'M50.11', 'M50.120', 'M50.121', 'M50.122', 'M50.123', 'M50.13', 'M50.20', 'M50.21', 'M50.220', 'M50.221', 'M50.222', 'M50.223', 'M50.23', 'M50.30', 'M50.31', 'M50.320', 'M50.321', 'M50.322', 'M50.323', 'M50.33', 'M50.80', 'M50.81', 'M50.820', 'M50.821', 'M50.822', 'M50.823', 'M50.83', 'M50.90', 'M50.91', 'M50.920', 'M50.921', 'M50.922', 'M50.923', 'M50.93', 'M53.0', 'M53.1', 'M53.2X1', 'M53.2X2', 'M53.2X3', 'M53.81', 'M53.82', 'M53.83', 'M54.01', 'M54.02', 'M54.03', 'M54.11', 'M54.12', 'M54.13', 'M54.2', 'M95.3', 'M99.01', 'M99.11', 'M99.31', 'M99.51', 'M99.61', 'M99.71', 'M99.81', 'Q76.411', 'Q76.412', 'Q76.413', 'S13.0XXA', 'S13.100A', 'S13.100D', 'S13.101A', 'S13.101D', 'S13.101S', 'S13.111A', 'S13.111D', 'S13.111S', 'S13.120A', 'S13.121A', 'S13.121D', 'S13.121S', 'S13.130A', 'S13.131A', 'S13.131D', 'S13.131S', 'S13.140A', 'S13.141A', 'S13.141D', 'S13.141S', 'S13.150A', 'S13.151A', 'S13.151D', 'S13.151S', 'S13.160A', 'S13.160D', 'S13.161A', 'S13.161D', 'S13.161S', 'S13.170D', 'S13.171A', 'S13.171D', 'S13.171S', 'S13.180A', 'S13.181A', 'S13.181D', 'S13.181S',

		'S13.20XA', 'S13.20XD', 'S13.20XS', 'S13.29XA', 'S13.29XD', 'S13.29XS', 'S13.4XXA', 'S13.4XXD', 'S13.4XXS', 'S13.5XXA', 'S13.8XXA', 'S13.8XXD', 'S13.8XXS', 'S13.9XXD', 'S13.9XXS', 'S16.1XXA', 'S16.1XXD', 'S16.1XXS') ('250.60', '250.61', '250.62', '250.63', '357.2', 'E08.42', 'E10.40', 'E10.42', 'E10.43', 'E10.610', 'E11.40', 'E11.42', 'E11.43', 'E11.610', 'E13.40', 'E13.42', '053.12', '053.13', '337.0', '337.00', '337.09', '337.1', '350', '350.1', '350.8', '350.9', '352.1', '353.8', '353.9', '354.8', '354.9', '355.79', '355.8', '355.9', '356.4', '356.8', '356.9', '729.2', 'B02.22', 'B02.23', 'E10.41', 'E13.41', 'E13.43', 'G50.0', 'G50.8', 'G50.9', 'G52.1', 'G54.8', 'G54.9', 'G56.80', 'G56.81', 'G56.82', 'G56.83', 'G56.90', 'G56.91', 'G56.92', 'G56.93', 'G57.80', 'G57.81', 'G57.82', 'G57.83', 'G57.90', 'G57.91', 'G57.92', 'G57.93', 'G58.7', 'G58.8', 'G58.9', 'G59', 'G60.2', 'G60.3', 'G60.8', 'G60.9', 'G61.9', 'G62.0', 'G62.89', 'G62.9', 'G63', 'G90.09', 'G99.0', 'M79.2') ('952', '952.0', '952.00', '952.01', '952.02', '952.03', '952.04', '952.05', '952.06', '952.07', '952.08', '952.09', '952.1', '952.10', '952.11', '952.12', '952.13', '952.14', '952.15', '952.16', '952.17', '952.18', '952.19', '952.2', '952.3', '952.4', '952.8', '952.9', 'S14.0XXA', 'S14.0XXD', 'S14.0XXS', 'S14.101A', 'S14.101D', 'S14.101S', 'S14.102A', 'S14.102D', 'S14.102S', 'S14.103A', 'S14.103D', 'S14.103S', 'S14.104A', 'S14.104D', 'S14.104S', 'S14.105A', 'S14.106A', 'S14.107A', 'S14.108A', 'S14.109A', 'S14.111A', 'S14.112A', 'S14.113A', 'S14.114A', 'S14.115A', 'S14.116A', 'S14.117A', 'S14.118A', 'S14.119A', 'S14.119D', 'S14.119S', 'S14.121A', 'S14.122A', 'S14.123A', 'S14.124A', 'S14.125A', 'S14.126A', 'S14.127A', 'S14.128A', 'S14.129A', 'S14.129D', 'S14.129S', 'S14.131A', 'S14.132A', 'S14.133A', 'S14.134A', 'S14.135A', 'S14.136A', 'S14.137A', 'S14.138A', 'S14.139A', 'S14.139D', 'S14.139S', 'S14.151A', 'S14.152A', 'S14.153A', 'S14.154A', 'S14.155A', 'S14.156A',

		'S14.157A', 'S14.158A', 'S24.0XXA', 'S24.0XXD', 'S24.0XXS', 'S24.101A', 'S24.101D', 'S24.101S', 'S24.102A', 'S24.102D', 'S24.102S', 'S24.103A', 'S24.104A', 'S24.109A', 'S24.111A', 'S24.112A', 'S24.113A', 'S24.114A', 'S24.119A', 'S24.119D', 'S24.119S', 'S24.131A', 'S24.132A', 'S24.133A', 'S24.134A', 'S24.139A', 'S24.139D', 'S24.139S', 'S24.151A', 'S24.152A', 'S24.153A', 'S24.154A', 'S34.01XA', 'S34.01XD', 'S34.01XS', 'S34.02XA', 'S34.02XD', 'S34.02XS', 'S34.109A', 'S34.111A', 'S34.111D', 'S34.111S', 'S34.112A', 'S34.112D', 'S34.112S', 'S34.113A', 'S34.113D', 'S34.113S', 'S34.114A', 'S34.114D', 'S34.114S', 'S34.115A', 'S34.115D', 'S34.115S', 'S34.119A', 'S34.119D', 'S34.119S', 'S34.121A', 'S34.121D', 'S34.121S', 'S34.122A', 'S34.122D', 'S34.122S', 'S34.123A', 'S34.123D', 'S34.123S', 'S34.124A', 'S34.124D', 'S34.124S', 'S34.125A', 'S34.125D', 'S34.125S', 'S34.129A', 'S34.129D', 'S34.129S', 'S34.131A', 'S34.131D', 'S34.131S', 'S34.132A', 'S34.132D', 'S34.132S', 'S34.139A', 'S34.139D', 'S34.139S', 'S34.3XXA', 'S34.3XXD', 'S34.3XXS') ('625.2', '625.3', '625.4', 'N94.0', 'N94.3', 'N94.4', 'N94.5', 'N94.6') ('625.7', '625.70', '625.71', '625.79', 'N94.810', 'N94.818', 'N94.819') ('595.1', 'N30.10', 'N30.11') ('617.0', '617.1', '617.2', '617.3', '617.4', '617.5', '617.6', '617.8', '617.9', 'N80.0', 'N80.1', 'N80.2', 'N80.3', 'N80.4', 'N80.5', 'N80.6', 'N80.8', 'N80.9')
	Cancer: identified using clinical stop codes. Veterans with a history of >2 visits to oncology clinics are considered patients who treat for cancer.	
	Severe Mental Illness after TBI ***index date*** *(including Schizophrenia, Bipolar Disorder, Psychosis)*:	('295', '295.01', '295.02', '295.03', '295.04', '295.05', '295.1', '295.11', '295.12', '295.13', '295.14', '295.15', '295.2', '295.21', '295.22', '295.23', '295.24', '295.25', '295.3', '295.31', '295.32', '295.33', '295.34', '295.35', '295.4', '295.41', '295.42', '295.43', '295.44', '295.45', '295.5', '295.51', '295.52', '295.53', '295.54',

		'295.55', '295.6', '295.61', '295.62', '295.63', '295.64', '295.65', '295.7', '295.71', '295.72', '295.73', '295.74', '295.9', '296', '296.01', '296.02', '296.03', '296.04', '296.05', '296.06', '296.1', '296.11', '296.12', '296.13', '296.14', '296.15', '296.16', '296.4', '296.41', '296.42', '296.43', '296.44', '296.45', '296.46', '296.5', '296.51', '296.52', '296.53', '296.54', '296.55', '296.56', '296.6', '296.61', '296.62', '296.63', '296.64', '296.65', '296.66', '296.7', '296.75', '296.8', '296.81', '296.89', '296.9', '296.99', '298.9', 'F06.0', 'F06.2', 'F20.0', 'F20.1', 'F20.2', 'F20.3', 'F20.5', 'F20.81', 'F20.89', 'F20.9', 'F22', 'F23', 'F24', 'F25.0', 'F25.1', 'F25.8', 'F25.9', 'F28.', 'F29.', 'F30.10', 'F30.11', 'F30.12', 'F30.13', 'F30.2', 'F30.3', 'F30.4', 'F30.8', 'F30.9', 'F31.0', 'F31.10', 'F31.11', 'F31.12', 'F31.13', 'F31.2', 'F31.30', 'F31.31', 'F31.32', 'F31.4', 'F31.5', 'F31.60', 'F31.61', 'F31.62', 'F31.63', 'F31.64', 'F31.70', 'F31.71', 'F31.72', 'F31.73', 'F31.74', 'F31.75', 'F31.76', 'F31.77', 'F31.78', 'F31.81', 'F31.89', 'F31.9', 'F53')
	Convulsions disorders,	ICD9: 345, 780.3 (with antiseizure medication, which I used VA drug class for ANTICONVULSANTS= CN400)<br>ICD10: G40.319, G40.419, G40.109, G40.111, G40.822, G40.41, G 40.401, G40.409.
	Medication-assisted treatment (MAT): We identified the MAT after the TBI index date using the algorithm provided by VA Pharmacy Benefits Management Services (Pharmacy Benefits Management Services, 2023).	If a patient with a history of OUD is on the prescription [a. Buprenorphine, b. Naltrexone tablet (for outpatient prescriptions, and CPRS orderable items), c. Naltrexone injection (for outpatient prescriptions, CPRS orderable items), d. Methadone] or outpatient procedures CPT Code 'J2315')] OR Outpatient visit to stop code for Visits to Opioid Treatment Program (OTP) ‘523’.
	Medications: We used VA drug classification codes to identify<br>1- antidepressants (including antidepressants, tricyclic antidepressants, monamine oxidase inhibitor antidepressants, antidepressants, other)	1- antidepressants (CN600, CN601, CN602, CN609)<br>2- opioids (CN101, RE301)<br>3- Antipsychotics (CN700, CN701, CN709)<br>4- Stimulants (CN800, CN801, CN802, CN809)

	2- opioids (opioid analgesics, opioid-containing antitussives/expectorants)<br>3- Antipsychotics (antipsychotics, phenothiazine/related antipsychotics, antipsychotics, other)<br>4- Stimulants (Central Nervous System stimulants, amphetamines, amphetamine like stimulants, CNS stimulants, other).<br>5- Hypnotics (no Benzodiazepine)= sedatives/hypnotics, barbituric acid derivative sedatives/hypnotics, sedatives/hypnotics, other<br>6- Benzodiazepine<br>7- Opioid Antagonist<br>8- Anticonvulsants	5- Hypnotics (no Benzodiazepine) = CN300, CN301, CN309<br>6- Benzodiazepine (CN302)<br>7- Opioid Antagonist (CN102)<br>8- Anticonvulsants (CN400)
**Covariates in the adjusted model**	*CUD, TBI, gender, age at the time of TBI, TBI severity, race, education, marital status, branch, rank, Rurality, service connected disability groups, District, Headache, Chronic Pain, MAT (recent), Oncology, SMI, Depression, PTSD, Personality Disorder, Alcohol Use Disorder, Opioid Use Disorder, Other SUD, Nicotine Use disorder, anxiety, insomnia, CHF, Perivascular disease, Cardiac disease, Stroke, DM, DM with complications, Convulsions disorders, Neurologic disorder (No Convulsions disorders), Liver Disease, and CKD.*	

Appendix References:

Austin, P.C. (2009a). Balance diagnostics for comparing the distribution of baseline covariates between treatment groups in propensity-score matched samples. *Stat Med* 28**,** 3083-3107.

Austin, P.C. (2009b). Using the Standardized Difference to Compare the Prevalence of a Binary Variable Between Two Groups in Observational Research. *Communications in Statistics - Simulation and Computation* 38**,** 1228-1234.

Elixhauser, A., Steiner, C., Harris, D.R., and Coffey, R.M. (1998). Comorbidity measures for use with administrative data. *Med Care* 36**,** 8-27.

Hai, T., Agimi, Y., and Stout, K. (2023). Prevalence of Comorbidities in Active and Reserve Service Members Pre and Post Traumatic Brain Injury, 2017-2019. *Mil Med* 188**,** e270-e277.

Kennedy, E., Panahi, S., Stewart, I.J., Tate, D.F., Wilde, E.A., Kenney, K., Werner, J.K., Gill, J., Diaz-Arrastia, R., Amuan, M., Van Cott, A.C., and Pugh, M.J. (2022). Traumatic Brain Injury and Early Onset Dementia in Post 9-11 Veterans. *Brain Inj* 36**,** 620-627.

Nuckols, T.K., Escarce, J.J., and Asch, S.M. (2013). The effects of quality of care on costs: a conceptual framework. *Milbank Q* 91**,** 316-353.

Ranganathan, P., Pramesh, C.S., and Buyse, M. (2015). Common pitfalls in statistical analysis: Clinical versus statistical significance. *Perspect Clin Res* 6**,** 169-170.

VA Health Care System, Pharmacy Benefits Management Services. (2023). https://www.pbm.va.gov/PBM/AcademicDetailingService/Opioid_Overdose_Education_and_Naloxone_Distribution.asp.
